# Improving the efficacy of plant-made anti-HIV monoclonal antibodies for clinical use

**DOI:** 10.3389/fpls.2023.1126470

**Published:** 2023-02-27

**Authors:** Melanie Grandits, Clemens Grünwald-Gruber, Silke Gastine, Joseph F. Standing, Rajko Reljic, Audrey Y-H. Teh, Julian K-C. Ma

**Affiliations:** ^1^ Molecular Immunology Unit, Institute for Infection and Immunity, St. George's University of London, London, United Kingdom; ^2^ Core Facility Mass Spectrometry, University of Natural Resources and Life Sciences, Vienna, Austria; ^3^ Infection, Immunity and Inflammation Research and Teaching Department, University College London (UCL) Great Ormond Street Institute of Child Health, London, United Kingdom

**Keywords:** bNAb, HIV, Half-Life, glycosylation, pharmacokinetics, antibody engineering, ADCC, plant molecular biopharming

## Abstract

**Introduction:**

Broadly neutralising antibodies are promising candidates for preventing and treating Human Immunodeficiency Virus/Acquired Immunodeficiency Syndrome (HIV/AIDS), as an alternative to or in combination with antiretroviral therapy (ART). These mAbs bind to sites on the virus essential for virus attachment and entry, thereby inhibiting entry into the host cell. However, the cost and availability of monoclonal antibodies, especially combinations of antibodies, hampers implementation of anti-HIV bNAb therapies in low- to middle- income countries (LMICs) where HIV-1 prevalence is highest.

**Methods:**

We have produced three HIV broadly neutralizing antibodies (bNAbs), 10-1074, VRC01 and 3BNC117 in the Nicotiana benthamiana transient expression system. The impact of specific modifications to enhance potency and efficacy were assessed. To prolong half-life and increase bioavailability, a M252Y/S254T/T256E (YTE) or M428L/N434S (LS) mutation was introduced. To increase antibody dependent cellular cytotoxicity (ADCC), we expressed an afucosylated version of each antibody using a glycoengineered plant line.

**Results:**

The majority of bNAbs and their variants could be expressed at yields of up to 47 mg/kg. Neither the expression system nor the modifications impacted the neutralization potential of the bNAbs. Afucosylated bNAbs exhibit enhanced ability to bind to FcγRIIIa and trigger ADCC, regardless of the presence of Fc amino acid mutations. Lastly, we demonstrated that Fc-modified variants expressed in plants show enhanced binding to FcRn, which results in a favourable in vivo pharmacokinetic profile compared to their unmodified counterparts.

**Conclusion:**

Tobacco plants are suitable expression hosts for anti-HIV bNAbs with increased efficacy and an improved pharmacokinetic profile.

## Introduction

Around 38 million people are living with Human Immunodeficiency Virus/Acquired Immunodeficiency Syndrome (HIV/AIDS) globally, with the majority of patients in less developed regions (UNAIDS, 2019). Combination antiretroviral therapy (cART) offers HIV patients the prospect of almost normal life-expectancy and very low risk of transmission (Mills et al., 2011). Yet, there are approximately 1.5 million new infections each year and still a considerable number of deaths (approximately 650000 people worldwide in 2021)(UNAIDS, 2021). Moreover, a 2019 report published by the WHO revealed that in 12 out of 18 assessed countries, drug resistance to first line non-nucleoside reverse transcriptase inhibitors (NNRTI) had exceeded 10% (World Health Organization, 2019). This trend was further emphasised by a survey conducted in nine sub-Saharan African countries, which showed that over 50% of infants newly diagnosed with HIV carry a NNRTI resistant strain (World Health Organization, 2019). Therefore, there is an urgent need for alternative treatment approaches.

Broadly neutralising antibodies (bNAbs) are considered to be one of the most promising candidates. They naturally develop approximately 1 year post-infection in rare individuals known as elite neutralisers (Doria-Rose et al., 2009). A number of bNAbs have been isolated, but only a few have been considered as treatment candidates for HIV-1. bNAbs 10-1074 (Mouquet et al., 2012), VRC01 (Wu et al., 2010) and 3BNC117 (Scheid et al., 2012) are three of the most promising candidates currently in clinical trials. Co-development of multiple bNAbs is necessary, as any bNAb-based therapy is likely to require administration of two or more antibodies to provide adequate coverage and avoid development of viral resistance (Caskey et al., 2016).

10-1074 targets the V3 loop and glycan on gp120 (Mouquet et al., 2012), which is responsible for binding to the co-receptor CCR5 or CXCR4. 10-1074 has a neutralisation breadth of about 60% and a half-life of 24 days in healthy individuals (Mouquet et al., 2012; Caskey et al., 2017). On the other hand, VRC01 and 3BNC117 target the CD4 binding site of gp120 (Wu et al., 2010; Scheid et al., 2012), which is essential for HIV-1 to enter the host cell. VRC01 has a neutralisation breadth of about 91% and 3BNC117 of 82%, with half-lives of 15 and 17 days respectively, in uninfected individuals (Caskey et al., 2015; Gaudinski et al., 2018).

Several *in vivo* studies have demonstrated the ability of bNAbs to protect against HIV-1 infection upon repeated exposure (Pegu et al., 2014; Gautam et al., 2016, 2018). Furthermore, there is evidence that a single course of early bNAb combination therapy can induce long-lasting virus control, as shown in Simian-Human Immunodeficiency Virus (SHIV) infected non-human primates (NHPs) treated with 3BNC117 and 10-1074 (Nishimura et al., 2017). Human clinical studies have demonstrated that VRC01, 3BNC117 and 10-1074 are well-tolerated and safe (Caskey et al., 2015, 2017; Ledgerwood et al., 2015), and there are ongoing studies investigating whether these bNAbs alone or in combination can be used to treat patients with established infection (e.g. NCT03571204, NCT02591420).

An affordable manufacturing platform is key to successful delivery of mAb-based therapies. About 93% of mAb production sites are located in either Europe or the USA (Grilo and Mantalaris, 2019), as low-to-middle income countries (LMICs) usually lack the capital to invest in traditional pharmaceutical production sites (Murad et al., 2020). Plants may offer an attractive alternative to current manufacturing platforms, as initial investment for upstream processes would be greatly reduced compared to mammalian-cell expression systems (Nandi et al., 2016) and culture medium – soil and fertiliser – is inexpensive and can be sourced locally (Murad et al., 2020). Furthermore, the cultivation of plants does not require highly specialised personnel and production can be modularly upscaled by expanding greenhouse facilities or acreage (Pogue et al., 2010). Successful expression of several anti-HIV bNAbs has been demonstrated in *Nicotiana benthamiana* and *Nicotiana tabacum*, with yields up to 400 mg/kg (Teh et al., 2014; Rosenberg et al., 2015), and plant-produced anti-HIV bNAb 2G12 has been shown to be safe and well-tolerated in humans (Ma et al., 2015).

bNAbs also have some limitations. bNAbs have to be given intravenously, whereas cART can be conveniently self-administered orally (Caskey et al., 2016). This would complicate bNAb therapy, especially in areas with limited medical infrastructure. Furthermore, monoclonal antibody therapies are expensive (average annual cost: $96,731) (Hernandez et al., 2018), thus frequent interventions with mAbs would add to the economic burden. This makes the case for increasing the potency of bNAbs and prolonging their *in vivo* half-lives, to reduce treatment frequency, improve patient compliance and reduce cost.

While the potential to effectively neutralise the virus is considered to be the main attribute of anti-HIV bNAbs, several studies have shown that their ability to perform effector functions, such as antibody-dependent cellular cytotoxicity (ADCC), can contribute to their potency and efficacy (Hessell et al., 2007, 2009; Bournazos et al., 2014). Moreover, it is speculated that bNAbs with the propensity for enhanced ADCC may be able to contribute to clearance of latent virus reservoirs (Caskey et al., 2016). The removal of the core fucose residue on the N297 glycan of Immunoglobulin Gs (IgGs) is one way to improve binding of the IgG Fc-region to FcγRIIIa, the key receptor that participates in ADCC (Vidarsson et al., 2014). Afucosylation of the N297 glycan has been shown to improve ADCC in mAbs targeting various diseases (Zeitlin et al., 2011).

Here, we use an emerging plant based expression system building on earlier clinical trials using plant-derived mAbs (Ma et al., 2015). IgG mAbs produced in plants differ significantly only in *N*-glycosylation compared with those produced in mammalian (CHO/HEK) cell-based systems. For this reason we used a glycoengineered plant line (ΔXF) (Strasser et al., 2008) for antibody production, in which fucosylation is minimised and glycoforms that are not normally produced by mammalian cells are avoided.

A key player in extending the half-life of IgGs is the neonatal receptor (FcRn), which salvages IgG from degradation in the lysosome (Petkova et al., 2006). Improvement in half-life is linked to increased affinity of IgG to FcRn at pH 6, which can be achieved by introducing a modification, for example M252Y/S254T/T256E (YTE) (Dall'Acqua et al., 2002) or M428L/N434S (LS) (Zalevsky et al., 2010) into the Fc region of the target antibody. These modifications resulted in a 3-4 fold enhancement of half-life of mAbs in NHPs and this approach has been widely used to prolong half-life of various IgGs (Dall'Acqua et al., 2002; Zalevsky et al., 2010; Gaudinski et al., 2018; Gautam et al., 2018). Currently several clinical studies are investigating the safety and efficacy of anti-HIV bNAbs carrying half-life extending mutations (e.g. NCT04250636, NCT05612178, NCT04173819).

We investigated the feasibility of using tobacco plants to express bNAbs, which have been modified to enhance half-life. These modifications include amino acid modifications (YTE or LS). We compared non-modified and modified versions of the anti-HIV bNAbs VRC01, 3BNC117 and 10-1074, with regard to their expression yield, *in vitro* functionality and *in vivo* half-life. We showed that neither the expression system nor the modifications impact neutralisation breadth and potency of the bNAbs. Furthermore, all plant-generated bNAbs exhibit higher affinity to FcγRIIIa, associated with removal of core-fucose, compared to their respective mammalian-cell made counterparts. This translated to higher ADCC activation *in vitro*. bNAbs with half-life extending modifications in their Fc-region showed improved binding to FcRn, which resulted in functional changes, demonstrated by *in vitro* transcytosis and most importantly up to 28% reduction in clearance in transgenic hFcRn mice when compared to the non-modified versions.

## Results

Here, we present the detailed data for bNAb 10-1074 only. Similar approaches and analyses were performed for bNAbs VRC01 and 3BNC117 and these are presented as supplemental figures and summarised at the end of this results section. If not specified, modified bNAb refers to the mutation affecting FcRn binding affinity in the Fc region.

### Expression of bNAbs in glycomodified *Nicotiana benthamiana* plants

YTE or LS mutations were introduced into the constant region of human γ-chain by site-directed mutagenesis. The respective variable regions of mAbs VRC01, 3BNC117 or 10-1074 were then introduced upstream of human light and heavy chain constant region genes. Three different versions of the heavy chain constant region were prepared: the non-modified (HC), YTE modified (YTE) or LS modified (LS) constant region. Heavy and light chain gene combinations were then assembled in MIDAS-p, a modular plant expression vector (Pinneh et al., 2021). All bNAbs were expressed transiently in ΔXF *Nicotiana benthamiana* (Strasser et al., 2008) using *Agrobacterium tumefaciens* harbouring the respective T-DNA constructs. The bNAb variants will be referred to as 10-1074 HC ΔXF, 10-1074 YTE ΔXF, 10-1074 LS ΔXF and compared with CHO-derived 10-1074 CHO.

*N. benthamiana* leaves were harvested 6 days post-infiltration (dpi). Average crude extract yields for 10-1074 HC ΔXF and 10-1074 YTE ΔXF were comparable (44.0 mg/kg ± 5.3 fresh weight (FW) vs 47.7 mg/kg ± 24.3 FW), whereas the average yield of 10-1074 LS was much lower (3.2 mg/kg 0.1 FW). The latter yields were considered below the threshold to generate reasonable purified amounts of this bNAb variant for further analysis, so this variant was abandoned.

A standard mAb purification protocol using Protein A affinity chromatography (Teh et al., 2014) resulted in recovery of 10-1074 HC ΔXF (13.3 ± 3.1 mg/kg FW) and of 10-1074 YTE ΔXF (9.2 ± 3.9 mg/kg FW), representing only 30% and 19% recovery respectively. Addition of 0.01% polysorbate 80 during affinity purification restored the recovery yield for 10-1074 HC ΔXF (47.0 ± 10.8 mg/kg FW) but had no impact on the yield of 10-1074 YTE ΔXF (12.6 ± 0.2 mg/kg FW).

### Integrity and purity of bNAb variants

**Figure 1:**
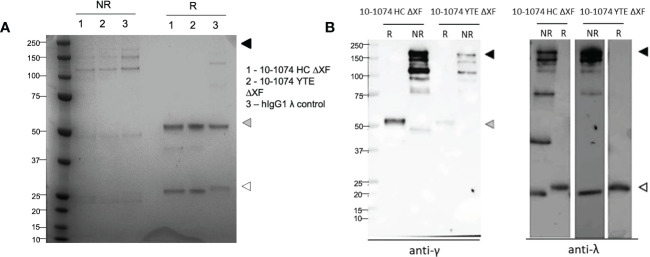
Yields and assembly of plant-expressed 10-1074 Fc variants. Representative SDS-PAGE **(A)** and anti-γ/Λ Western blots **(B)** of non-reduced (NR) and reduced (R) purified 10-1074 non-modified (HC) and YTE variant expressed in λXF *N. benthamiana* plants. Arrows indicate fully assembled bNAb (black), heavy chain (grey) or light chain (white). Commercial human IgG1λ from human myeloma plasma (Sigma, USA) was used as control.

The quality and purity of 10-1074 HC ΔXF and YTE ΔXF were confirmed by SDS-PAGE (**Figure 1A**). Under non-reducing conditions, bands corresponding to the fully assembled antibody (150-180 kDa) and minor degradation products were observed. The pattern of bands was similar to that of a commercial myeloma-derived human IgG mAb control. Under reducing conditions, bands consistent with intact heavy and light chains were detected. This was confirmed by western blot, which showed specific immunoreactivity for each band, with either anti-gamma or anti-lambda chain antisera (**Figure 1B**, right).

### Glycosylation of bNAb variants

**Figure 2:**
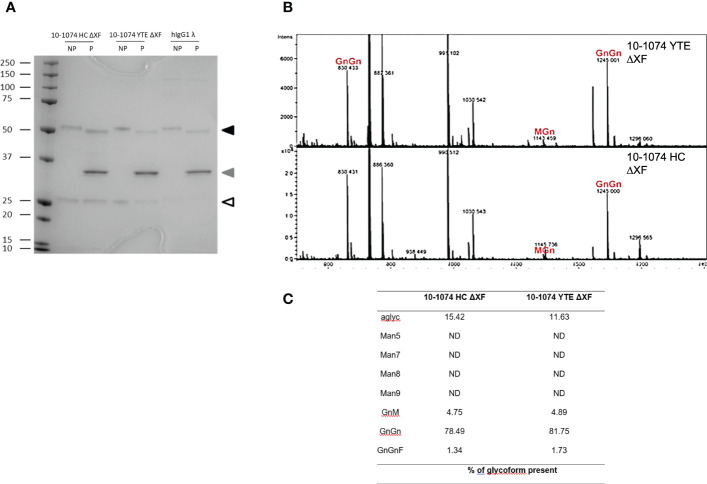
Glycosylation profiles of 10-1074 Fc variants. **(A)** Representative SDS-PAGE of untreated (NP) and PNGase F treated (P) 10-1074 HC ΔXF and YTE ΔXF variants, as well as mammalian cell-produced hIgG1λ. Arrows indicate enzymatically aglycosylated heavy chain (black), PNGase F (grey) and light chain (white). **(B, C)** Mass spectra showing dominant glycans present in purified 10-1074 YTE ΔXF (B; top) and 10-1074 HC ΔXF (B; bottom), as well as percentage of glycoforms present **(C)**. *N*-glycans are abbreviated according to the ProGlycAn system (www.proglycan.com).

bNAbs produced in ΔXF *Nicotiana benthamiana* were expected to lack α1,3-fucose and β1,2-xylose. PNGase F is an enzyme that cleaves all *N*-linked oligosaccharides from proteins unless the core-GlcNAc carries α1,3-fucose. bNAbs were digested with PNGase F and resolved using SDS-PAGE alongside undigested bNAb controls. For all digested samples expressed in ΔXF *N. benthamiana*, a size shift in the heavy chain compared to the undigested control could be observed (**Figure 2A** – black triangle), supporting the absence of core fucose glycosylation.

This was confirmed by mass spectrometry (**Figure 2B**), which not only revealed GnGn as the prevalent glycoform (~80%) of 10-1074 HC ΔXF and YTE ΔXF, but also confirmed the absence of β1,2-xylose and only low-levels of α1,3-fucose (<2%). Up to 15% of bNAb were aglycoslyated (**Figure 2C**).

### Binding kinetics to HIV-1 gp140

Surface plasmon resonance (SPR) was used to compare the HIV-1 UG37 gp140 binding affinity of the plant-produced bNAb variants and their mammalian-produced non-modified counterparts (**Supplementary Table 4**). No significant differences were observed (p>0.05, Tukey's multiple comparison test).

### HIV-1 neutralisation

**Figure 3:**
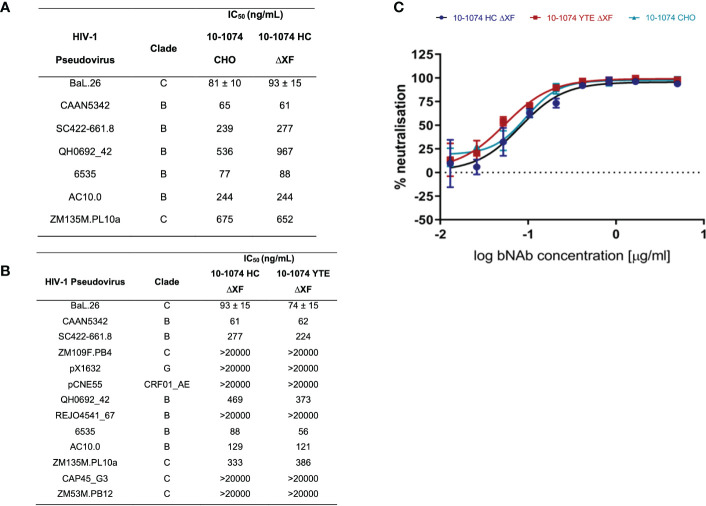
HIV-1 pseudovirus neutralisation comparison. **(A)** IC_50_s of 10-1074 HC ΔXF and 10-1074 CHO against seven HIV-1 pseudovirus strains susceptible to 10-1074. BaL.26 (n=3 biological repeats), all other pseudovirus strains (n=1). **(B)** IC_50_s of 10-1074 HC ΔXF and YTE ΔXF against 13 HIV-1 pseudovirus strains from different clades. Mean ± SD for BaL.26 (n=3 biological repeats) are shown. For all other pseudovirus strains n=1. **(C)** BaL.26 neutralisation curves of ΔXF *N. benthamiana* plant-made 10-1074 HC (blue), YTE (red), and 10-1074 CHO (light blue). Data points indicate average ± SD of 3 technical repeats.

Viral neutralisation of bNAb variants was measured against a panel of HIV-1 pseudoviruses (**Figure 3**). First, we confirmed that the plant expressed 10-1074 HC ΔXF had equivalent neutralising activity compared to 10-1074 CHO, using a panel of seven HIV isolates (**Figure 3A**). A more extensive comparison was then performed, comparing 10-1074 HC ΔXF with 10-1074 YTE ΔXF, using a panel of 13 HIV isolates (**Figure 3B**). No differences were observed. Comparative neutralisation curves are also shown for the three antibodies against HIV BaL.26 (**Figure 3C**).

### Binding kinetics to FcγRIIIa (CD16a) and ADCC

**Figure 4:**
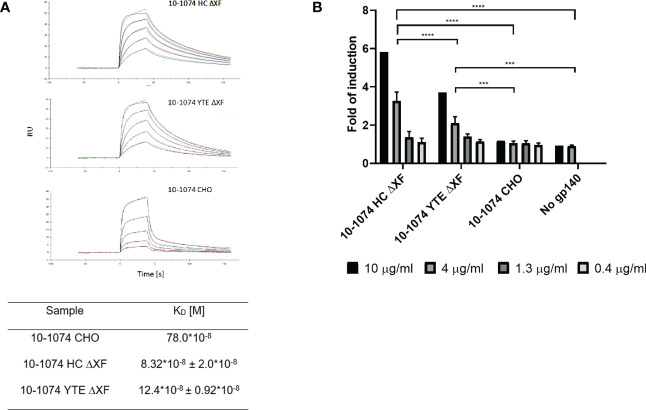
FcγRIIIa binding and ADCC activation comparison. **(A)** Representative SPR sensorgram showing binding of ΔXF *N. benthamiana*-produced 10-1074 HC ΔXF (top), 10-1074 YTE ΔXF (middle) and mammalian cell-produced 10-1074 CHO (bottom) to FcγRIIIa V158 at five different concentrations (0.0625, 0.125, 0.25, 0.5 and 1 μM) along with equilibrium dissociation constants (K_D_s; bottom table). KDs of 10-1074 HC ΔXF and YTE ΔXF were averages ± SD of three biological repeats. **(B)** FcγRIIIa activation by 10-1074 HC ΔXF, YTE ΔXF and CHO in the presence of HIV-1 UG37 gp140. Activation was measured by fold-induction of luciferase expression controlled by NFAT pathway. Values represent average fold-induction ± SD of 3 biological repeats (except for 10 μg/mL, where n=1). A two-way ANOVA with Tukey's multiple comparison test was performed to determine significant differences. 10-1074 HC ΔXF without HIV-1 UG37 gp140 was used as negative control. Significant differences could only be observed at 4 μg/ml (ns p > 0.05, ***p≤ 0.001, ****p ≤ 0.0001).

Binding kinetics of the plant-produced 10-1074 variants to soluble FcγRIIIa V158 were compared to their mammalian counterpart using SPR. Both bNAb variants produced in glycomodified ΔXF plants showed improved binding (9.4 and 6.3-fold respectively) when compared to their glycan unmodified, fucosylated mammalian counterpart (**Figure 4A**)

To determine whether increased affinity of the plant-made bNAbs to FcγRIIIa V158 results in a functional improvement in antibody-dependent cellular cytotoxicity (ADCC), a Reporter Assay was performed with effector cells carrying FcγRIIIa V158 (**Figure 4B**). For both ΔXF plant-made bNAbs, a concentration-dependent induction was observed. At 4 and 10μg/ml, 10-1074 HC ΔXF and YTE ΔXF induced significantly higher ADCC activation compared with the CHO produced 10-1074 mAb (p<0.05). At these concentrations, 10-1074 HC ΔXF was also significantly more potent than 10-1074 YTE ΔXF (p<0.05).

### Binding kinetics to FcRn and *in vitro* transcytosis

**Figure 5:**
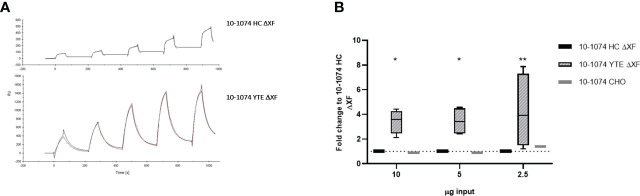
hFcRn binding and *in vitro* transcytosis comparison. **(A)** SPR sensorgrams showing binding of 10-1074 HC ΔXF (top) and 10-1074 YTE ΔXF plant-produced 10-1074 (bottom) to hFcRn at five different concentrations (75, 100,150, 200 and 250 nM). **(B)** Transcytosis of 10-1074 HC ΔXF, YTE ΔXF and CHO across MDCK.2 cell layer constitutively expressing hFcRn/hβ_2_m. Values indicate average fold-change ± SD of bNAbs in output wells (pH 7.4) compared to 10-1074 HC ΔXF when 2.5, 5 or 10 μg of bNAbs were introduced into the input wells (pH 6.0). hβ_2_m expressing MDCK.2 cell layers were used as negative controls. 4 biological repeats were performed for plant-made 10-1074 HC ΔXF and YTE ΔXF and one for 10-1074 CHO. A two-way ANOVA with Tukey's multiple comparison test with 10-1074 HC ΔXF as control was performed to determine significant differences (*p≤ 0.05, **p≤ 0.01).

Binding of bNAbs bearing the YTE mutation to human neonatal Fc receptor (hFcRn) was determined by SPR. CM5 chips were coated directly with the 10-1074 bNAb variants and a series of concentrations of recombinant hFcRn were flowed over the chip. The sensorgrams show much higher binding of recombinant hFcRn to 10-1074 YTE ΔXF when compared to the HC ΔXF non-modified control (**Figure 5A**). Whilst an affinity of 1.021 x 10^-7^ M could be calculated for 10-1074 YTE ΔXF, the unusual nature of the dissociation curve for HC ΔXF made it impossible to calculate a comparative value.

To demonstrate that this change in binding affinity to FcRn had a functional consequence, a transcytosis assay was performed using transgenic MDCK.2 cells constitutively expressing hFcRn/hβ_2_m. MDCK.2 hβ_2_m-expressing cells served as control. Transepithelial resistance was measured to verify the formation of tight junctions and ranged between 250-350 Ω*cm^2^. bNAbs were added to the apical side (pH 6.0) and the basolateral supernatant was removed after 2 hours of incubation to determine the amount of transcytosed bNAb by ELISA. Increased transcytosis was observed at all concentrations for 10-1074 YTE ΔXF. Minimal or no antibody was detected in the basolateral supernatant when 10-1074 HC ΔXF or 10-1074 CHO was tested. (**Figure 5B**).

### *In vivo* pharmacokinetic studies

**Figure 6:**
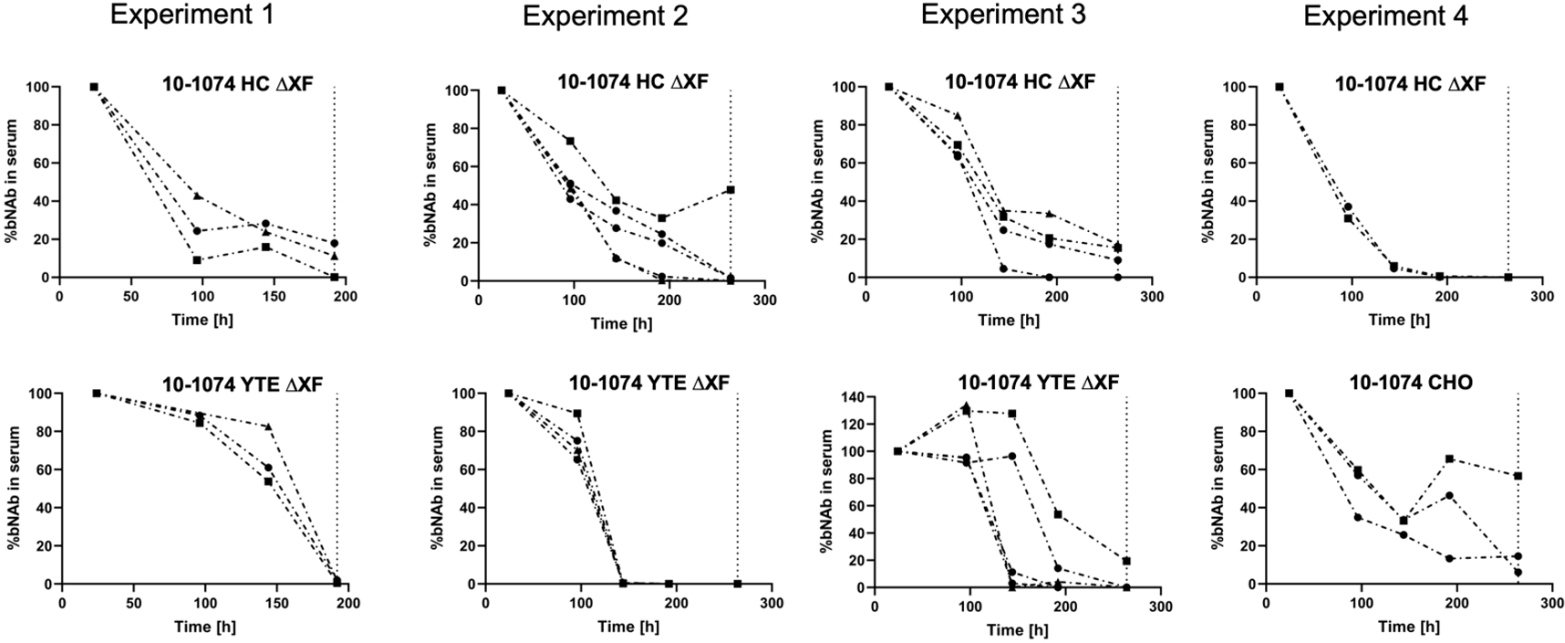
*In vivo* pharmacokinetics of 10-1074 Fc variants. Serum concentration (%) of 10-1074 HC ΔXF, YTE ΔXF and CHO in hFcRn transgenic mice across four separate experiments measured using ELISA. Serum concentration at day one was set to 100%. Each line represents a separate animal. The dashed vertical line demonstrates timepoint of sacrifice.

*In vivo* pharmacokinetic studies were performed using B6.Cg-*Fcgrt^tm1Dcr^* Tg(CAG-FCGRT)276Dcr/DcrJ mice transgenic for hFcRn, that were given a single intravenous 2 mg/kg dose of bNAb. Results from four replicate experiments are shown (**Figure 6**). 10-1074 CHO and 10-1074 HC ΔXF followed first-order pharmacokinetics (**Figure 6**, Expt. 4 and Expts. 1-3 top graphs, **Supplementary Figure 7**). 10-1074 YTE ΔXF antibody depletion was delayed, following a nonlinear pattern most likely attributed to target mediated drug disposition kinetics (Dua et al., 2015). In Expts 1-3, 5/12 mice retained an average of 84.4% of the bNAb at 144 hours **Figure 6** lower graphs), compared with an average of 24.7% in the 10-1074 HC ΔXF inoculated mice.

The area-under-the-curve (AUC, %*h) was calculated (**Supplementary Table 5**), as basic determination of half-life cannot be performed due to the mixed linear and nonlinear kinetics of 10-1074 YTE. The AUC showed that the introduction of the YTE mutation led to a 39% increase in AUC and therefore a reduction in clearance of 10-1074.

### bNAbs VRC01 and 3BNC117

The average post-purification yield for VRC01 and 3BNC117 was in both cases highest for the non-modified (HC) version of the bNAb at 65 ± 8 mg/kg FW and 51 ± 21 mg/kg FW, respectively (**Supplementary Figure 1A**). The post-purification yield of VRC01 YTE ΔXF was 39mg/Kg, VRC01 LS ΔXF was 33mg/Kg, 3BNC117 YTE ΔXF was 31mg/Kg and 3BNC117 LS ΔXF was 38mg/Kg. Batch-to-batch variability was higher in plants infiltrated with YTE or LS constructs.

All bNAbs were assembled correctly (**Supplementary Figures 1B–D**) and PNGase digest verified the absence of α1,3-fucose in all variants of both bNAbs (**Supplementary Figures 2A and B**). As both VRC01 and 3BNC117 contain a glycosylation site in the light chain, a size shift in the light chain could also be observed for these samples (**Supplementary Figures 2A and B**).

A slight reduction in HIV gp140 binding affinity was determined for plant produced VRC01 but not for 3BNC117 mAb (**Supplementary Table 2**). This was not however, supported by decreased neutralisation potency of the plant-produced bNAbs (**Supplementary Table 3**), as observed in previous studies (Rosenberg et al., 2013; Teh et al., 2014). As seen with 10-1074, modified variants of both bNAbs performed equally in neutralisation assay when compared to the respective non-modified version (**Supplementary Table 3**).

Binding kinetics of all variants to FcγRIIIa V158 showed a 2.7 – 15-fold increase in affinity of the glycomodified plant-produced bNAbs compared to the non-modified, fucosylated mammalian versions. Like 10-1074, this increase is highest for the HC (Fc-unmodified) version of both bNAbs (1.6-4.9x) when compared to the Fc-modified bNAbs (**Supplementary Figure 3A**), but all variants demonstrated increased ADCC pathway activation (**Supplementary Figures 3B and C**).

Binding kinetics of VRC01 and 3BNC117 bNAbs to hFcRn was measured using CM5 chips coated with an anti-Human IgG Fab reagent. Equilibrium dissociation constants (K_D_s) were in the 10^-8^M range (**Supplementary Figure 4A**). The YTE and LS versions of VRC01 and 3BNC117 had the highest affinities to hFcRn (**Supplementary Figure 4A**). Transcytosis assays showed that YTE and LS versions of the bNAbs was more efficiently transcytosed through hFcRn expressing cells (**Supplementary Figures 4B and C**).

*In vivo*, the decline in antibody after intravenous administration followed first-order kinetics (**Supplementary Figure 5A**). As with 10-1074, the modified versions demonstrated a favourable pharmacokinetic profile when compared to the non-modified versions. However, for VRC01 the mammalian produced version showed a more favourable profile than any of the plant-produced versions in the initial experiment. The AUC of VRC01 YTE ΔXF and VRC01 LS ΔXF was 35% and 28% higher respectively than VRC1 HC ΔXF. It was not possible to determine pharmacokinetics for 3BNC117 in the Tg276 mouse model, as bNAb levels were almost depleted after 96 h (**Supplementary Figure 5B**).

## Discussion

bNAbs are of increasing interest as adjuncts or alternatives to cART for prevention or treatment of HIV-1 infection (Caskey et al., 2016), and are being assessed in a number of clinical trials. This is driven by the lack of a vaccine and the emergence of resistance to first line NNRTI which has been observed in up to 26% of ART initiators (World Health Organization, 2019). Additionally, passive immunisation with bNAbs may help with the reduction of the latent reservoir and trigger the immune system to effectively fight the virus (Caskey et al., 2016; Nishimura et al., 2017; Desikan et al., 2019). However, while the concept of bNAbs as sole treatment or in combination with cART seems promising, the need for intravenous administration and cost are major disadvantages (Caskey et al., 2016).

Improving efficacy by prolonging half-life and enhancing effector functions would be an important step to making bNAbs more accessible and affordable, particularly in countries with limited infrastructure. We also propose that a plant-based mAb expression platform may facilitate this goal. Here we investigated whether anti-HIV bNAbs with enhanced properties can be generated in glyco-engineered tobacco plants without compromising their potency. To improve efficacy, we introduced an YTE or LS mutation into the Fc-region, both known to prolong half-life *in vivo*. bNAbs with these improved properties may eventually allow for implementation of bNAbs for the treatment of chronically infected patients, and potentially be useful as pre- and post-exposure prophylaxis by offering immediate and effective long-term protection with less side-effects than current treatments. Furthermore, treatments with bNAbs may be beneficial in HIV-positive pregnant women, where standard Mother-to-Child Prevention therapy is either ineffective or initiated too late. As well as providing direct anti-viral treatment to the mother, placental transport of IgG *via* FcRn is a route for infants to obtain protective maternal IgGs *in utero* (Simister, 2003).

Three anti-HIV-1 bNAbs were successfully expressed in ΔXF *N. benthamiana* plants at high yields. Engineered versions of these antibodies were also produced, targeting the Fc region to alter binding to FcRn. Although there appears to be some yield penalty, variant bNAbs were still expressed at relatively high levels, with the exception of the LS mutant of bNAb 10-1074. For 10-1074 there was also a reduction in antibody recovery after Protein A affinity purification. This could be mitigated in the non-mutated heavy chain antibody version by the addition of Tween 80, but not in the YTE mutant. While Tween 80 is commercially used to protect mAbs from interface-induced aggregation, it is possible that the polysorbate may not be able to fully 'protect' mAbs carrying a YTE mutation, which has shown to lead to an around 11% loss in thermodynamic stability (Edgeworth et al., 2015). All plant-produced bNAb variants retained specificity and affinity to their antigen and displayed similar neutralisation potency compared with the same antibodies expressed conventionally in CHO cells. This is in accordance with previous reports showing that potency was not affected by whether the bNAb was produced in a mammalian-cell or plant platform (Teh et al., 2014; Rosenberg et al., 2015; Gautam et al., 2018). Furthermore, our data confirmed that neither afucosylation nor the introduction of the YTE or LS mutation had any effect on the neutralisation potential of the bNAbs. This result was to be expected, as all alterations were limited to the Fc-region, even though some studies have suggested that changes in the Fc-region can influence the functionality of the Fab domain and vice versa (Cooper et al., 1994; Igawa et al., 2010; Tudor et al., 2012; Schoch et al., 2015; Jensen et al., 2017).

Afucosylation of the core-GlcNAc of mAbs has previously been shown to result in increased affinity to FcγRIIIa, which translated into enhanced ADCC activity (Shields et al., 2002; Okazaki et al., 2004; Ferrara et al., 2011; Luo et al., 2017; Marusic et al., 2018; Pereira et al., 2018; Stelter et al., 2020). We corroborated these findings and demonstrated that an increase in affinity to FcγRIIIa, and the subsequent improved induction of ADCC, can be obtained by removal of the N297 fucose in the presence of a YTE or LS mutation. The capability to perform ADCC may be important for maximising the potency of anti-HIV bNAbs – a number of studies have demonstrated a reduction in protection against HIV-1 conferred by bNAbs upon abrogation of effector functions (Hessell et al., 2007, 2009; Bournazos et al., 2014). Furthermore, several studies have shown a correlation between slow disease progression and high ADCC activity of antibodies (Baum et al., 1996; Banks et al., 2002; Gómez-Román et al., 2005; Lambotte et al., 2013; Madhavi et al., 2017), suggesting that bNAbs with enhanced ADCC may be beneficial for virus control.

The magnitude of improvement in ADCC varied between the three tested bNAbs, even though they had identical Fc-regions with differences only in the variable region. This supports findings of a recent study investigating the interaction of FcγRIIIa and mAbs, which revealed that several positions in the Fab-region, including the variable region, may be involved in the interaction with FcγRIIIa (Yogo et al., 2019). Interestingly, our results suggest that the Fab region could determine whether mutations introduced in the Fc region negatively affect the bNAb's potential to induce ADCC. There was no change in ADCC pathway activation between the different variants of 3BNC117 and VRC01, whereas 10-1074 YTE ΔXF showed a statistically significant reduced induction. Reduced ADCC ability of mAbs with YTE mutations has been previously reported (Dall''Acqua et al., 2006; Ko et al., 2014).

Introduction of the YTE or LS mutation led in all cases to improved binding to the neonatal receptor at pH 6 without impacting release at pH 7.4, consistent with previous reports for other mAbs (Dall'Acqua et al., 2002; Zalevsky et al., 2010). The higher affinity to the FcRn translated functionally to enhanced *in vitro* transcytosis. Transcytosis potential has been used as an indicator of *in vivo* clearance for mAbs engineered for altered binding to the neonatal receptor (Jaramillo et al., 2017).

Higher affinity for the FcRn resulted in a favourable pharmacokinetic profile, as has been established before for several mammalian-cell produced mAbs (Dall'Acqua et al., 2002; Zalevsky et al., 2010; Ko et al., 2014; Gautam et al., 2018). For 10-1074 and VRC01, we showed that the plant-produced bNAbs carrying the YTE and LS mutation result in a 26-28% and 22% reduction in *in vivo* clearance, respectively, in hFcRn Tg276 mice compared to the plant-produced non-modified version. These improvements are comparable to data from previous studies investigating half-life extension in the Tg276 hFcRn mouse model (Kang et al., 2018; Valente et al., 2020). Unfortunately, pharmacokinetics could not be determined for 3BNC117 with the employed sampling protocol, as the antibody was rapidly depleted after 96 h. While the pharmacokinetic data of hFcRn mice correlates with profiles seen in NHPs and humans (Tam et al., 2013; Avery et al., 2016), the half-lives of the same mAbs in Tg276 hFcRn transgenic mice may be up to 4.5 times shorter than in NHPs (Valente et al., 2020). In the case of 10-1074 YTE ΔXF a sudden, rapid decline in serum bNAb levels between 96 and 144 h was observed in half of the mice. Rosenberg et al. (2019) reported a similar drop in NHPs, when PGT121 – also a V3 glycan dependent bNAb – was modified with YTE. The authors established that the observed rapid decline correlated with the development of anti-drug antibodies against the bNAb (Rosenberg et al., 2019). However, in our study, IgM levels at 196 h and 264 h did not differ between those showing a rapid decline and those that did not (**Supplementary Figure 6**, Methods S1). Another reason for these differences in pharmacokinetics may have been the poor condition of the Tg276 offspring after several breeding rounds, leading to breeding difficulties which have been reported before (Tam et al., 2013). The offspring showed failure to thrive and could only be partially improved by a specialised diet. It is possible that hFcRn expression levels may vary in mice with impacted health, though it was not possible to confirm this hypothesis retrospectively. Thus the results from Experiment 2 and 3, which were performed with mice with impacted health, may be less reliable than the results obtained from Experiment 1. For future studies of pharmacokinetics of human mAbs it would be preferable to use the well-established Tg32 (B6.Cg-*Fcgrt^tm1Dcr^* Tg(FCGRT)32Dcr/DcrJ) strain or potentially the newly established SYNB-h*FCRN* strain (Conner et al., 2022).

In conclusion, we have demonstrated that tobacco plants are suitable expression hosts for anti-HIV bNAbs modified by glycoengineering to provide enhanced efficacy in terms of binding to FcγRIIIa (for ADCC); and by YTE or LS mutations to enhance binding to FcRn, translating to enhanced transcytosis, *in vivo* half-life and slower clearance from the circulation (**Supplementary Table 6**). Importantly, we identified 10-1074 YTE and VRC01 LS in ΔXF *N. benthamiana* as lead candidates for further development. The production of these antibodies in plants would broaden the potential applications of bNAbs used as a one-time treatment in specific circumstances, such as prevention of mother-to-child-transmission late in pregnancy, post-exposure prophylaxis, and other emergency procedures where rapid control of reduction of viral load is necessary. Further work to assess the production of anti-HIV bNAbs in plants at scale is now warranted, to follow up on recent regulatory approvals and successful clinical trials for plant derived biologics (Ma et al., 2015; Ward et al., 2021).

## Materials and methods

### Control antibodies produced in mammalian cell expression systems

Mammalian cell-produced 10-1074 (ARP12477), 3BNC117 (ARP12474) and VRC01 (ARP3291) were obtained from the AIDS Reagent Programme through the Centre for AIDS Reagents, NIBSC, UK.

### Cloning of antibody heavy and light chain genes into plant expression (MIDAS-P) vectors

Site-directed mutagenesis was performed using QuikChange II site-directed mutagenesis kit (Agilent, USA) along with primers (**Supplementary Table 1**) designed to introduce the M252Y/S254T/T256E (YTE) or M428L/N434S (LS) mutations into human IgG1 Fc region with BsaI sites to insert variable regions. 10-1074 (Mouquet et al., 2012), VRC01 (Wu et al., 2010) and 3BNC117 (Scheid et al., 2011) light and heavy chain variable regions (V_L_ and V_H_) were synthesised with BsaI sites and cloned into vectors containing modified or non-modified constant regions of human IgG1 heavy chain (C_H_), human IgG lambda chain (C_L_; for 10-1074), or human IgG kappa chain (C_L_; for VRC01, 3BNC117). The MIDAS-P system (Pinneh et al., 2021) was used for tandem expression of the heavy and light chain genes in a single binary vector. Briefly, the heavy and light chain genes were cloned into the MIDAS-p entry vectors pWhite and pBlue (Pinneh et al., 2021) respectively. Then, pWhite or pBlue vectors containing the genes of interests were assembled into the destination vector pMIDAS using Golden Gate Assembly with BsaI (for pWhite) and then BsmBI (for pBlue). All plant expression vectors were then electroporated into *Agrobacterium tumefaciens* strain GV3101 PMP90/RK.

### Agrobacterium transformation of *N. benthamiana* plants using vacuum infiltration

Vacuum infiltration of glycomodified ΔXF line (Strasser et al., 2008) or wild-type (WT) *N. benthamiana* was performed as described by Kapila et al. (1997). *Agrobacterium tumefaciens* GV3101/PMPRK carrying the heavy and light chain in MIDAS-p for each bNAb was grown for 24 h in Luria-Bertani broth supplemented with 50 µg/mL rifampicin, kanamycin and carbenicillin. After centrifugation the bacterial pellet was resuspended in infiltration solution containing 0.1 mM acetosyringone, 0.01 mM MES and 0.01 mM MgCl_2_. The final OD_600_ of the suspension was adjusted to 0.2. 7-week-old ΔXF *Nicotiana benthamiana* plants were immersed into the bacterial suspension in a desiccator attached to a vacuum pump, a vacuum was applied for 2 minutes at 150 mbar before releasing, allowing the bacterial solution into the interstitial spaces of the plant leaves. The plants were then further grown in a controlled environment at 26°C for 6 days with a 18/6 hr light/dark cycle.

### Extraction and purification of bNAbs from plant leaves

Antibodies were extracted from infiltrated leaves 6 days post infiltration (dpi) in 3 volumes (w/v) of PBS pH 7.4 with or without addition of 0.01% of polysorbate 80. Plant debris was removed by filtration through miracloth and centrifugation at 15008 x *g* for 50 minutes at 4°C. The crude extract was passed through a 0.45 µm filter, followed by a 0.22 µm filter.

mAbs were purified using Protein A-agarose (Sigma Aldrich, USA). The column was calibrated with 5 column volumes (CVs) of Binding Buffer (Sodium phosphate pH 7.0), before the introduction of the clarified plant crude extract. Washing steps were performed using 10 CVs of Binding Buffer and elution was performed using 0.1 M Glycine-HCl (pH 2.7). The eluate was neutralised using 1 in 10 dilution of 1 M Tris-HCl (pH 9.0) with or without addition of 0.1% polysorbate 80. Buffer-exchange to PBS (pH 7.4) was carried out using a Slide-A-Lyzer Dialysis Cassette (3500 MWCO). An Amicon Ultra-15 Centrifugal filter was used to concentrate the sample approximately 10-fold. Purified antibody concentrations were determined using Surface Plasmon Resonance.

The purity and assembly of antibodies was verified using SDS-PAGE and Western blot respectively. Samples were separated using NuPAGE 4-12% Bis-Tris gels (Novex, Invitrogen, USA) with 1% MOPS buffer (Novex, Invitrogen, USA). Gels were stained with Instant Blue (Expedeon, UK). For Western blotting, the separated proteins were blotted onto a nitrocellulose membrane (Amersham, GE Healthcare, UK) by semi-dry transfer. Blocking was performed with 5% (w/v) non-fat dried milk (NFDM) powder (Marvel) in TBST [1% (v/v) TBS + 0.1% (v/v) Tween 20]. Anti-hIgG-γ or anti-hIgG-κ/λ (both from The Binding Site, UK) antiserum was used for detection of heavy and light chains, respectively. The blots were developed using Pierce ECL Plus Western Blotting detection system following the manufacture's protocol.

### PNGase F digest

PNGase F digest was carried out following the manufacturer's instructions (New England Biolabs, USA). Digested and non-digested glycoproteins were then separated *via* SDS-PAGE using a NuPAGE 10% Bis-Tris gel (Novex, Invitrogen, USA).

### HIV-1 neutralisation assay

Neutralisation potency of the generated bNAbs was determined using a TZM-bl assay and HIV-1 pseudovirus as previously described (Wei et al., 2003; Montefiori, 2004; Sarzotti-Kelsoe et al., 2014). All cells were grown in and dilutions performed using Growth Medium [DMEM with 10% Fetal Calf Serum (FCS), Penicillin-Streptomycin (100 units/mL and 100µg/mL respectively)]. Pseudoviruses were generated by transfecting HEK 293T cells with an *Env*-deficient backbone plasmid and the relevant *Env*-plasmid.

For neutralisation assays, bNAbs were used at a starting concentration of 5 μg/ml and titrated threefold in triplicate in clear flat-bottom 96-well plates. Pseudoviruses at a dilution 20x above the background was added to each well, excluding the cells-only control, and incubated for 1 h. 1x10^4^ TZM-bl cells (Centre for AIDS Reagents, NIBSC, UK), in media supplemented with DEAE dextran, were added to each well and plates were incubated for 48 h at 37°C, 5% CO_2_. The supernatant was removed, cells were washed with PBS and lysis buffer (Promega, USA) was added and plates were kept at -80°C overnight. The thawed cell lysate was mixed 1:1 with Bright-Glo luciferase substrate (Promega Luciferase Assay System, USA) in a black flat bottom 96 well plate. Luminescence was measured using a GloMax plate reader (96 Microplate Luminometer, Promega, USA). Percentage of reduction in relative light units (RLU) was calculated relative to the RLU of the positive (virus plus cells) control. GraphPad Prism was used to plot the resulting curve and calculate IC_50_s.

### Surface plasmon resonance (SPR)

Antibody concentration was determined by capturing the purified antibodies and titrations of known concentration of human IgG kappa (Sigma, UK) on the Protein A-CM5 chip. The response units (RUs) were used to calculate antibody concentration from a standard curve using the BIAcore Evaluation software.

The binding kinetics of all variants of 10-1074, 3BNC117 and VRC01 to gp140, FcγRIIIa and FcRn were determined using SPR on a BIAcore X-100 instrument (Cytiva, USA) at 25°C. Measurements for gp140 and FcγRIIIa were performed using a Protein A capture approach. Protein A (Sigma Aldrich, USA) was immobilised onto a CM5 chip aiming for 5000 response units (RU) with standard amine coupling. HBS-EP+ (10 mM HEPES, pH7.4, 150 mM NaCl, 3 mM EDTA and 0.05% surfactant P-20) was used as running buffer. Each bNAb was captured to a R_max_ of 50. For gp140 measurements, UG37 gp140 (HIV Reagent Programme, USA) was applied at a concentration of 80 μg/ml and a flow rate of 40 μL/min. A contact time of 135s and a dissociation time of 3600s were used. Binding kinetics to FcγRIIIa were determined by applying a range of FcγRIIIa (R&D Systems, USA) concentrations (1, 0.5, 0.25, 0.125 and 0.0625 μM). A contact time of 40s and a flow rate of 50 μL/min were used. Chips were regenerated with 10 mM glycine-HCl, pH 1.5 for both experiments.

The binding kinetics of hFcRn to 10-1074 variants was determined by coating CM5 chips with the respective bNAb to a RU of 10000 and applying multiple concentrations (75,100,150,200,250 nM) of rhFcRn (R&D Systems, USA) at a flow rate of 30 µL/min, a contact time of 60s and dissociation time of 90s. Binding of VRC01 and 3BNC117 to FcRn was determined by using a Human Fab Capture kit (Cytiva, UK). The bNAbs were captured to the Rmax of 100RU and rhFcRn at concentrations of 10, 25, 50,75,100 nM were flowed over the chip with contact time of 60s and dissociation time of 90s, at a flow rate of 30 µL/min. In both experiments, PBS-T (PBS with 0.05% Tween-20), pH 6.0 was used as running buffer. Chips were regenerated with 10 mM glycine-HCl, pH 2.1.

All sensorgrams were corrected with appropriate blank references and fit globally with BIAcore Evaluation software using a 1:1 Langmuir model of binding or a two-state reaction model.

### Transcytosis assay

Human neonatal Fc receptor (hFcRn) transcytosis assay was performed as previously described (Claypool et al., 2002). Briefly, 0.75 x 10^6^ MDCK hβ_2_m (vector only control) or MDCK hFcRn/hβ_2_m (supplied by Professor Richard Blumberg of Harvard Medical School) cells were grown on 12mm transwell plates with a pore size of 0.4µm (CoStar, USA) in Growth Medium [DMEM with 10% Fetal Calf Serum (FCS), Penicillin-Streptomycin (100 units/mL and 100µg/mL respectively) and 2mM L-glutamine] at 37°C, 5% CO_2_ for 4 days. 12 hours before the experiments, the medium was changed to serum-free medium without antibiotics. Trans-epithelial resistance was measured on the day of the experiment. The cells in the transwell were washed with Hanks' Balanced Salt Solution (HBSS), pH 7.4. Then, the input (apical) and output (basal) wells were equilibrated with HBBS, pH 6.0 and HBBS, pH 7.4 respectively, for 20 minutes, at 37°C, 5% CO_2_. 10, 5 or 2.5 µg of respective bNAbs were added to apical side (pH 6). Basolateral supernatant was collected after a 2 hour further incubation. Antibody concentration was quantified using ELISA.

### Enzyme-linked immunosorbent assay (ELISA)

ELISA was used to detect mAbs in transcytosis assays and for serum half-life experiments. 96-well flat-bottom immunosorbent plates were coated overnight at 4°C with 5 μg/ml of sheep α-hIgG γ-chain antibody (The Binding Site, UK). Wells were blocked with 5% NFDM (w/v) in PBS with 0.1% (v/v) Tween 20 for 1 hour at 37°C.

Serum samples were applied at 1:50 (Bleed 1) and 1:15 (all remaining bleeds) dilution in duplicate. Basolateral supernatants from transcytosis assays were applied without dilution. A threefold serial dilution row in blocking buffer was performed. Plates were incubated overnight at 4°C. Peroxidase-conjugated anti-IgG κ or IgG λ (Sigma Aldrich, USA) at 1 in 1000 in blocking buffer was applied and plates incubated for 1 h at 37°C. Plates were washed with wash buffer (water with 0.01% Tween 20) after each incubation step. Bound antibodies were detected using TMB substrate solution (Invitrogen, USA). The colour reaction was stopped using 2M sulphuric acid and absorbance was measured at 450nm using a Sunrise plate reader (Tecan). Antibody concentrations were determined using titration curves of known human IgG κ or IgG λ as standards.

### Antibody-dependent cellular cytotoxicity (ADCC) activation assay

To determine the ability of the bNAbs to activate ADCC, ADCC Bioreporter Assay, V Variant (Promega, USA) was used. bNAbs were diluted to an appropriate starting concentration and 3-fold dilution series performed in sterile white flat bottom 96 well plates. An equal volume of HIV-1 UG37 gp140 was added to each well and plates incubated for 1 hour at 37 (5% CO_2_). ADCC effector cells (Jurkat T cells constitutively expressing surface FcγRIIIa-V158) were added to each well. Plates were incubated for 6 hours at 37°C, 5% CO_2_, then, left at room temperature for 20 minutes before adding Bio-Glo Luciferase Substrate (Promega, USA). After 5 minutes, luminescence was measured using a GloMax. ADCC pathway activation was calculated as fold induction [Fold of induction= RLU (sample-substrate)/RLU(cells only-substrate)].

### *In vivo* pharmacokinetic studies in human neonatal Fc receptor (hFcRn) transgenic mice

C57BL/6 mice with the mouse neonatal Fc receptor (FcRn) knocked out and transgenic for human FcRn [mFcRn KO hFcRn(276) ^Tg/Tg^; stock number 004919, The Jackson Laboratories, USA], were housed and bred in the Biological Research Facilities (BRF) at St. George's University of London (SGUL) under Home Office Regulations. All animal experiments were reviewed and approved by the Animal Welfare and Ethical Review Body (AWERB) of SGUL.

7- to 9-week-old male and female mice were randomised based on age and weight. Animal number per group (3-5) was chosen in accordance with previously published papers (Petkova et al., 2006; Zalevsky et al., 2010). Mice were weighed two days prior to injection. bNAbs were injected intravenously *via* the tail vein at 2mg/kg (~30-60 µg/mouse). PBS was injected as negative control. 50 µL blood samples were taken from the tail or saphenous vein after 24, 96, 144, 192 hours post-infusion. Mice were sacrificed after 192 or 264 h and a final blood sample *via* cardiac puncture was taken.

Blood samples were allowed to clot for 1 hour at 37°C followed by 4°C overnight, before centrifugation at 13,000rpm for 10 minutes and collection of serum. Serum was kept at -20°C. Analysis of bNAb present in serum was performed *via* an α-hIgG ELISA. The percentage of antibodies remaining in the serum was calculated against that from day 1 (set as 100%). The AUC was calculated using GraphPad Prism.

### Statistics

All graphs were drawn and analysed using the GraphPad prism 8 software (GraphPad, USA). A One-way or two-way ANOVA with Tukey's multiple comparison test was carried out for statistical analysis as indicated in the text and/or figure legends.

## Data availability statement

The raw data supporting the conclusions of this article will be made available by the authors, without undue reservation.

## Ethics statement

The animal study was reviewed and approved by St George's Animal Welfare and Ethical Review Body (AWERB) and conducted at Biological Research Facility, under the project licence P1A7411AD.

## Author contributions

Conceptualisation and design of study: AY-HT and JK-CM. Experimental investigation: MG, CG-G, and AY-HT. Data analysis: MG, SG, JS, JK-CM, and AY-HT. Writing (original draft): MG. Animal licence: RR. Funding acquisition: JK-CM. All authors contributed to the article and approved the submitted version.

## Supplementary material

Supplementary Table 1 Primers used in site-directed mutagenesis to introduce M252Y/S254T/T256E (YTE) or M428L/N434S (LS) mutation in heavy (γ) chain Fc regions of VRC01 or 3BNC117/10-1074. Nucleotides in bold indicate changes in the sequence. A different primer pair was chosen for VRC01 as a plasmid was already available and the nucleotide sequence of the Fc region -while maintaining the same amino acid composition- differed slightly to the one used for 3BNC117 and 10-1074.

Supplementary Table 2 Equilibrium dissociation constant (K_D_), association constant (K_a_) and dissociation constant (K_d_) of VRC01 and 3BNC117 with unmodified (HC) or modified Fc regions (YTE or LS) to HIV-1 UG37 gp140. Values were from one biological repeat. VRC01 or 3BNC117 produced in HEK cells were used as positive control.

Supplementary Table 3 IC_50_s of HC ΔXF, YTE ΔXF and LS ΔXF versions of VRC01 or 3BCN117 against 5 HIV-1 pseudovirus strains from different clades. Mean ± SD for BaL.26 (n=3 biological repeats) are shown. VRC01 or 3BNC117 produced in HEK cells were used as positive control.

Supplementary Table 4 Equilibrium dissociation constant (K_D_), association constant (K_a_) and dissociation constant (K_d_) of 10-1074 Fc variants, as well as CHO-produced 10-1074 to HIV-1 UG37 gp140. Values for plant-made 10-1074 were the averages ± SD of 3 biological repeats, while values for 10-1074 CHO were from one measurement.

Supplementary Table 5 Mean ± SD of area under the curve (AUC) (%*h) calculated for 10-1074 HC ΔXF, YTE ΔXF and CHO (n= number per group).

Supplementary Table 6 Summary table of results for all variants of the 3 bNAbs 10-1074, VRC01 and 3BNC117. Highest average achieved yield is shown (with addition of 0.01% polysorbate 80 for 10-1074 HC and YTE ΔXF). For transcytosis fold change (compared the matching HC ΔXF) is only shown for the highest input (10 μg) of the respective bNAb.

Supplementary Figure 1
**(A)** Post-purification yields (expressed as mg/kg leaf fresh weight) of the unmodified **(HC)**, M252Y/S254T/T256E **(YTE)** or M428L/N434S **(LS)** variant of anti-HIV-1 bNAb VRC01 and 3BNC117. All bNAbs were expressed in glycomodified *Nicotinana benthamiana* plants lacking core β1,2-xylose and α1,3-fucose (ΔXF). Values are the average ± SD of 3 biological repeats. **(B)** Representative SDS-PAGE **(B)** of non-reduced **(NR)** and reduced **(R)** purified VRC01 HC, YTE or LS expressed in ΔXF *N. benthamiana* plants. Arrows indicate fully assembled bNAb (black), heavy chain (white) or light chain (grey). VRC01 from HEK cells was used as positive control. **(C and D)** Representative Western blot of non-reduced **(NR; C)** or SDS-PAGE of reduced **(R; D)** purified 3BNC117 HC, YTE or LS expressed in ΔXF *N. benthamiana* plants. Arrows indicate fully assembled bNAb (black), heavy chain (white) or light chain (grey). 3BCN117 from HEK cells was used as positive control for Western blot.

Supplementary Figure 2 Representative SDS-PAGE showing deglycosylation of ΔXF VRC01 **(A)** and 3BNC117 **(B)** Fc variants after PNGase treatment. NP indicate untreated and P indicated PNGase F treated samples. Arrows indicated enzymatically aglycosylated heavy chain (black), PNGase F (grey) and light chain (white).

Supplementary Figure 3
**(A)** Equilibrium dissociation constants (K_D_s) of ΔXF *N. benthamiana*-produced HC, YTE, LS Fc variants and mammalian cell-produced HEK HC variant of anti-HIV bNAbs VRC01 and 3BNC117 to FcγRIIIa. B and C) FcyRIIIa activation by ΔXF HC, ΔXF YTE, ΔXF LS variants, as well as wild type *N. benthamiana* **(WT)**-made HC and HEK HC variants of VRC01 **(B)** and 3BCN117 **(C)** in the presence of HIV-1 UG37 gp140. Activation was measured by fold-induction of luciferase expression controlled by NFAT pathway. Values represent average fold-induction ± SD of 2 biological repeats. A two-way ANOVA with Tukey's multiple comparison test was performed to determine significant differences.

Supplementary Figure 4
**(A)** Equilibrium dissociation constants (K_D_s) of ΔXF *N. benthamiana*-produced HC, YTE, LS Fc variants and mammalian cell-produced HC HEK variant of VRC01 and 3BNC117 to human neonatal Fc receptor (hFcRn). **(B and C)** Transcytosis of ΔXF *N. benthamiana-*made HC, YTE, LS, as well as HC HEK variants of VRC01 **(B)** and 3BCN117 **(C)** across MDCK.2 cell layer constitutively expressing hFcRn/hβ_2_m. hβ_2_m-expressing MDCK.2 cell layers were used as negative controls. pH of input wells and output wells were pH6 and 7.4 respectively. Values represent average ± SD of 2 biological repeats.

Supplementary Figure 5
**(A)** Serum concentration of VRC01 ΔXF HC, ΔXF YTE, ΔXF LS and HEK HC in hFcRn transgenic mice measured using ELISA. Each line represents a separate animal. Mean ± SD of area under the curve (AUC) (%*h) calculated for VRC01 ΔXF HC, ΔXF YTE, ΔXF LS and HEK HC (n= number per group) are shown. **(B)** Serum concentration of 3BNC117 ΔXF HC, ΔXF YTE, ΔXF LS and HEK HC in hFcRn transgenic mice using ELISA. Each value represents average ± SD of 3-4 animals.

Supplementary Figure 6 Individual values, mean ± SD of relative standardised serum anti-hIgG murine IgM levels for all experiments. IgM level differences were not statistically significant (p≥0.05) between 10-1074 variants.

Supplementary Figure 7 Log10 serum concentration (%) of 10-1074 HC ΔXF, YTE ΔXF and CHO in hFcRn transgenic mice across four separate experiments highlighting the linear elimination pattern of 10-1074 HC ΔXF and 10-1074 CHO and mixed-mode of 10-1074 YTE ΔXF. Data points for zero percent values cannot be transformed into log10 and are consequently missing in the graph.

## References

[B1] Avery L. B. Wang M. Kavosi M. S. Joyce A. Kurz J. C. Fan Y. Y.. (2016). Utility of a human FcRn transgenic mouse model in drug discovery for early assessment and prediction of human pharmacokinetics of monoclonal antibodies. MAbs 8 (6), 1064–78. doi: 10.1080/19420862.2016.1193660 27232760 PMC4968115

[B2] Banks N. D. Kinsey N. Clements J. Hildreth J. E. (2002). Sustained antibody-dependent cell-mediated cytotoxicity (ADCC) in SIV-infected macaques correlates with delayed progression to AIDS. AIDS Res Hum Retroviruses 18 (16), 1197–1205. doi: 10.1089/08892220260387940 12487826

[B3] Baum L. L. Cassutt K. J. Knigge K. Khattri R. Margolick J. Rinaldo C.. (1996). HIV-1 gp120-specific antibody-dependent cell-mediated cytotoxicity correlates with rate of disease progression. J. Immunol. 157 (5), 2168–2173. 8757343

[B4] Bournazos S. Klein F. Pietzsch J. Seaman M. S. Nussenzweig M. C. Ravetch J. V. (2014). Broadly neutralizing anti-HIV-1 antibodies require fc effector functions for *In vivo* activity. Cell 158 (6), 1243–1253. doi: 10.1016/j.cell.2014.08.023 25215485 PMC4167398

[B5] Caskey M. Klein F. Lorenzi J. C. Seaman M. S. West A. P. Jr Buckley N.. (2015). Viraemia suppressed in HIV-1-infected humans by broadly neutralizing antibody 3BNC117. Nature 522 (7557), 487–491. doi: 10.1038/nature14411 25855300 PMC4890714

[B6] Caskey M. Schoofs T. Gruell H. Settler A. Karagounis T. Kreider E. F.. (2017). Antibody 10-1074 suppresses viremia in HIV-1-infected individuals. Nat. Med. 23 (2), 185–191. doi: 10.1038/nm.4268 28092665 PMC5467219

[B7] Caskey M. Klein F. Nussenzweig M. C. (2016). Broadly neutralizing antibodies for HIV-1 prevention or immunotherapy. N. Engl. J. Med. 375 (21), 2019–2021. doi: 10.1056/NEJMp1613362 27959740

[B8] Claypool S. M. Dickinson B. L. Yoshida M. Lencer W. I. Blumberg R. S.. (2002). Functional reconstitution of human FcRn in madin-Darby canine kidney cells requires co-expressed human β2-microglobulin. J. Biol. Chem. 277 (31), 28038–28050. doi: 10.1074/jbc.M202367200 12023961 PMC2825174

[B9] Conner C. M. van Fossan D. Read K. Cowley D. O. Alvarez O. Xu S.. (2022). A humanized fcrn transgenic mouse for preclinical pharmacokinetics studies. BioRxiv, 2022.10.24.513622. doi: 10.1101/2022.10.24.513622 36870576

[B10] Cooper L. J. Robertson D. Granzow R. Greenspan N. S. (1994). Variable domain-identical antibodies exhibit IgG subclass-related differences in affinity and kinetic constants as determined by surface plasmon resonance. Mol. Immunol. Engl. 31 (8), 577–584. doi: 10.1016/0161-5890(94)90165-1 7515151

[B11] Dall'Acqua W. F. Woods R. M. Ward E. S. Palaszynski S. R. Patel N. K. Brewah Y. A.. (2002). Increasing the affinity of a human IgG1 for the neonatal fc receptor: biological consequences. J. Immunol. 169 (9), 5171–5180. doi: 10.4049/jimmunol.169.9.5171. United States. 12391234

[B12] Dall’Acqua W. F. Kiener P. A. Wu H. (2006). Properties of human IgG1s engineered for enhanced binding to the neonatal fc receptor (FcRn). J. Biol. Chem. 281 (33), 23514–23524. doi: 10.1074/jbc.M604292200 16793771

[B13] Desikan R. Raja R. Dixit N. M. (2019). Early exposure to broadly neutralizing antibodies triggers a switch from progressive disease to lasting control of SHIV infection. bioRxiv p, 548727. doi: 10.1101/548727 PMC7462315 32817614

[B14] Doria-Rose N. A. Klein R. M. Manion M. M. O'Dell S. Phogat A. Chakrabarti B.. (2009). Frequency and phenotype of human immunodeficiency virus envelope-specific b cells from patients with broadly cross-neutralizing antibodies. J. Virol. 83 (1), 188–199. doi: 10.1128/jvi.01583-08 18922865 PMC2612342

[B15] Dua P. Hawkins E. van der Graaf P. H. (2015). A tutorial on target-mediated drug disposition (TMDD) models. CPT: Pharmacometrics Syst. Pharmacol. 4 (6), 324–337. doi: 10.1002/psp4.41 26225261 PMC4505827

[B16] Edgeworth M. J. Phillips J. J. Lowe D. C. Kippen A. D. Higazi D. R. Scrivens J. H. (2015). Global and local conformation of human IgG antibody variants rationalizes loss of thermodynamic stability. Angew Chem Int Ed Engl 54 (50), 15156–15159. doi: 10.1002/anie.201507223 26482340

[B17] Ferrara C. Grau S. Jäger C. Sondermann P. Brünker P. Waldhauer I.. (2011). Unique carbohydrate-carbohydrate interactions are required for high affinity binding between FcγRIII and antibodies lacking core fucose. Proc Natl Acad Sci USA. 108 (31), 12669–12674. doi: 10.1073/pnas.1108455108 21768335 PMC3150898

[B18] Gaudinski M. R. Coates E. E. Houser K. V. Chen G. L. Yamshchikov G. Saunders J. G.. (2018). Safety and pharmacokinetics of the fc-modified HIV-1 human monoclonal antibody VRC01LS: A phase 1 open-label clinical trial in healthy adults. PloS Med. 15 (1), e1002493. doi: 10.1371/journal.pmed.1002493 29364886 PMC5783347

[B19] Gautam R. Nishimura Y. Pegu A. Nason M. C. Klein F. Gazumyan A.. (2016). A single injection of anti-HIV-1 antibodies protects against repeated SHIV challenges. Nature 533 (7601), 105–109. doi: 10.1038/nature17677 27120156 PMC5127204

[B20] Gautam R. Nishimura Y. Gaughan N. Gazumyan A. Schoofs T. Buckler-White A.. (2018). A single injection of crystallizable fragment domain-modified antibodies elicits durable protection from SHIV infection. Nat Med. 24 (5), 610–616. doi: 10.1038/s41591-018-0001-2 29662199 PMC5989326

[B21] Gómez-Román V. R. Patterson L. J. Venzon D. Liewehr D. Aldrich K. Florese R.. (2005). Vaccine-elicited antibodies mediate antibody-dependent cellular cytotoxicity correlated with significantly reduced acute viremia in rhesus macaques challenged with SIV mac251. J. Immunol. 174 (4), 2185–2189. doi: 10.4049/jimmunol.174.4.2185 15699150

[B22] Grilo A. L. Mantalaris A. (2019). The increasingly human and profitable monoclonal antibody market. Trends Biotechnol. Elsevier Ltd 37 (1), 9–16. doi: 10.1016/j.tibtech.2018.05.014 29945725

[B23] Hernandez I. Bott S. W. Patel A. S. Wolf C. G. Hospodar A. R. Sampathkumar S.. (2018). Pricing of monoclonal antibody therapies: higher if used for cancer? Am J Manag Care. 24(2), 109–112. 29461857

[B24] Hessell A. J. Hangartner L. Hunter M. Havenith C. E. Beurskens F. J. Bakker J. M.. (2007). Fc receptor but not complement binding is important in antibody protection against HIV. Nature 449 (7158), 101–104. doi: 10.1038/nature06106 17805298

[B25] Hessell A. J. Poignard P. Hunter M. Hangartner L. Tehrani D. M. Bleeker W. K.. (2009). Effective, low-titer antibody protection against low-dose repeated mucosal SHIV challenge in macaques. Nat. Med. Nat. Publishing Group 15 (8), 951–954. doi: 10.1038/nm.1974 PMC4334439 19525965

[B26] Igawa T. Tsunoda H. Tachibana T. Maeda A. Mimoto F. Moriyama C.. (2010). Reduced elimination of IgG antibodies by engineering the variable region. Protein Engineering Design Selection 23 (5), 385–392. doi: 10.1093/protein/gzq009 20159773

[B27] Jaramillo C. A. C. Belli S. Cascais A. C. Dudal S. Edelmann M. R. Haak M.. (2017). Toward in vitro-to-in vivo translation of monoclonal antibody pharmacokinetics: Application of a neonatal Fc receptor-mediated transcytosis assay to understand the interplaying clearance mechanisms. MAbs 9 (5), 781–791. doi: 10.1080/19420862.2017.1320008 28440708 PMC5524153

[B28] Jensen P. F. Schoch A. Larraillet V. Hilger M. Schlothauer T. Emrich T.. (2017). A two-pronged binding mechanism of IgG to the neonatal fc receptor controls complex stability and IgG serum half-life. Mol. Cell. Proteomics 16 (3), 451–456. doi: 10.1074/mcp.M116.064675 28062799 PMC5341005

[B29] Kang C. Xia L. Chen Y. Zhang T. Wang Y. Zhou B.. (2018). A novel therapeutic anti-HBV antibody with increased binding to human FcRn improves *in vivo* PK in mice and monkeys. Protein Cell 9 (1), 130–134. doi: 10.1007/s13238-017-0438-y 28677103 PMC5777975

[B30] Kapila J. Rycke R. D. Montagu M. V. Angenon G.. (1997). An agrobacterium-mediated transient gene expression system for intact leaves. Plant Sci. 122 (1), 101–108. doi: 10.1016/S0168-9452(96)04541-4

[B31] Ko SY Pegu A Rudicell RS Yang ZY Joyce MG Chen X. (2014). Enhanced neonatal Fc receptor function improves protection against primate SHIV infection. Nature 514 (7524), 642–5. doi: 10.1038/nature13612 25119033 PMC4433741

[B32] Lambotte O. Pollara J. Boufassa F. Moog C. Venet A. Haynes B. F.. (2013). High antibody-dependent cellular cytotoxicity responses are correlated with strong CD8 T cell viral suppressive activity but not with B57 status in HIV-1 elite controllers. PloS One 8 (9), e74855. doi: 10.1371/journal.pone.0074855 24086385 PMC3781132

[B33] Ledgerwood J. E. Coates E. E. Yamshchikov G. Saunders J. G. Holman L. Enama M. E.. (2015). Safety, pharmacokinetics and neutralization of the broadly neutralizing HIV-1 human monoclonal antibody VRC01 in healthy adults. Clin. Exp. Immunol. 182 (3), 289–301. doi: 10.1111/cei.12692 26332605 PMC4636891

[B34] Luo C. Chen S. Xu N. Wang C. Sai W. B. Zhao W.. (2017). Glycoengineering of pertuzumab and its impact on the pharmacokinetic/pharmacodynamic properties. Sci. Rep. 7, 46347. doi: 10.1038/srep46347 28397880 PMC5387714

[B35] Madhavi V. Wines B. D. Amin J. Emery S. ENCORE1 Study Group Lopez E.. (2017). HIV-1 env- and vpu-specific antibody-dependent cellular cytotoxicity responses associated with elite control of HIV. J. virology United States 91 (18):e00700-17. doi: 10.1128/JVI.00700-17 PMC5571238 28701393

[B36] Ma JK Drossard J Lewis D Altmann F Boyle J Christou P. (2015). Regulatory approval and a first-in-human phase I clinical trial of a monoclonal antibody produced in transgenic tobacco plants. Plant Biotechnol. J. 13 (8), 1106–1120. doi: 10.1111/pbi.12416 26147010

[B37] Marusic C. Pioli C. Stelter S. Novelli F. Lonoce C. Morrocchi E.. (2018). N-glycan engineering of a plant-produced anti-CD20-hIL-2 immunocytokine significantly enhances its effector functions. Biotechnol Bioeng 115 (3), 565–576. doi: 10.1002/bit.26503 29178403

[B38] Mills E. J. Bakanda C. Birungi J. Chan K. Ford N. Cooper C. L.. (2011). Life expectancy of persons receiving combination antiretroviral therapy in low-income countries: A cohort analysis from Uganda. Ann. Internal Med. 155 (4), 209–217. doi: 10.7326/0003-4819-155-4-201108160-00358 21768555

[B39] Montefiori D. C. (2004). Evaluating neutralizing antibodies against HIV, SIV, and SHIV in luciferase reporter gene assays. Curr Protoc Immunol Chapter 12:12.11.1-12.11.17. doi: 10.1002/0471142735.im1211s64 18432938

[B40] Mouquet H. Scharf L. Euler Z. Liu Y. Eden C. Scheid J. F.. (2012). Complex-type n-glycan recognition by potent broadly neutralizing HIV antibodies. Proc Natl Acad Sci U S A. 109 (47), E3268–E3277. doi: 10.1073/pnas.1217207109 23115339 PMC3511153

[B41] Murad S. Fuller S. Menary J. Moore C. Pinneh E. Szeto T.. (2020). Molecular pharming for low and middle income countries’. Curr. Opin. Biotechnol. Elsevier Ltd, 61 (November 2019), 53–59. doi: 10.1016/j.copbio.2019.10.005 31751895

[B42] Nandi S. Kwong A. T. Holtz B. R. Erwin R. L. Marcel S. McDonald K. A. (2016). Techno-economic analysis of a transient plant-based platform for monoclonal antibody production. MAbs 8 (8), 1456–1466. doi: 10.1080/19420862.2016.1227901 27559626 PMC5098453

[B43] Nishimura Y. Gautam R. Chun T. W. Sadjadpour R. Foulds K. E. Shingai M.. (2017). Early antibody therapy can induce long-lasting immunity to SHIV. Nature 543 (7646), 559–563. doi: 10.1038/nature21435 28289286 PMC5458531

[B44] Okazaki A. Shoji-Hosaka E. Nakamura K. Wakitani M. Uchida K. Kakita S.. (2004). Fucose depletion from human IgG1 oligosaccharide enhances binding enthalpy and association rate between IgG1 and FcγRIIIa. J. Mol. Biol. 336 (5), 1239–1249. doi: 10.1016/j.jmb.2004.01.007 15037082

[B45] Pegu A. Yang Z. Y. Boyington J. C. Wu L. Ko S. Y. Schmidt S. D.. (2014). Neutralizing antibodies to HIV-1 envelope protect more effectively *in vivo* than those to the CD4 receptor. Sci. Trans. Med. 6 (243), 243ra88–243ra88. doi: 10.1126/scitranslmed.3008992 PMC4562469 24990883

[B46] Pereira N. A. Chan K. F. Lin P. C. Song Z.. (2018). The “less-is-more” in therapeutic antibodies: Afucosylated anti-cancer antibodies with enhanced antibody-dependent cellular cytotoxicity. MAbs 10 (5), 693–711.doi: 10.1080/19420862.2018.1466767 29733746 PMC6150623

[B47] Petkova S. B. Akilesh S. Sproule T. J. Christianson G. J. Al Khabbaz H. Brown A. C.. (2006). Enhanced half-life of genetically engineered human IgG1 antibodies in a humanized FcRn mouse model: potential application in humorally mediated autoimmune disease. Int. Immunol. 18 (12), 1759–1769. doi: 10.1093/intimm/dxl110 17077181

[B48] Pinneh E. C. van Dolleweerd C. J. Göritzer K. Drake P. M. W. Ma J. K. Teh A. Y. (2021). Multiple gene expression in plants using MIDAS-p, a versatile type II restriction-based modular expression vector. Biotechnol. Bioengineering. 119(6), 1660–1672. doi: 10.1002/bit.28073 PMC9313558 35238400

[B49] Pogue G. P. Vojdani F. Palmer K. E. Hiatt E. Hume S. Phelps J.. (2010). Production of pharmaceutical-grade recombinant aprotinin and a monoclonal antibody product using plant-based transient expression systems. Plant Biotechnol. J. 8 (5), 638–654. doi: 10.1111/j.1467-7652.2009.00495.x 20514694

[B50] Rosenberg Y. Sack M. Montefiori D. Forthal D. Mao L. Hernandez-Abanto S.. (2013). Rapid high-level production of functional HIV broadly neutralizing monoclonal antibodies in transient plant expression systems. PloS One 8 (3):e58724. doi: 10.1371/journal.pone.0058724 23533588 PMC3606348

[B51] Rosenberg Y. Sack M. Montefiori D. Labranche C. Lewis M. Urban L.. (2015). Pharmacokinetics and immunogenicity of broadly neutralizing HIV monoclonal antibodies in macaques. PloS One 10 (3), e0120451. doi: 10.1371/journal.pone.0120451 25807114 PMC4373669

[B52] Rosenberg Y. J. Lewis G. K. Montefiori D. C. LaBranche C. C. Lewis M. G. Urban L. A.. (2019). Introduction of the YTE mutation into the non-immunogenic HIV bnAb PGT121 induces anti-drug antibodies in macaques. PloS One 14 (2), e0212649. doi: 10.1371/journal.pone.0212649 30785963 PMC6437720

[B53] Sarzotti-Kelsoe M. Bailer R. T. Turk E. Lin C. L. Bilska M. Greene K. M.. (2014). Optimization and validation of the TZM-bl assay for standardized assessments of neutralizing antibodies against HIV-1. J. Immunol. Methods 409, 131–146. doi: 10.1016/j.jim.2013.11.022 24291345 PMC4040342

[B54] Scheid J. F. Mouquet H. Ueberheide B. Diskin R. Klein F. Oliveira T. Y.. (2011). Sequence and structural convergence of broad and potent HIV antibodies that mimic CD4 binding. Sci. (New York N.Y.) 333 (6049), 1633–1637. doi: 10.1126/science.1207227 PMC3351836 21764753

[B55] Schoch A. Kettenberger H. Mundigl O. Winter G. Engert J. Heinrich J.. (2015). Charge-mediated influence of the antibody variable domain on FcRn-dependent pharmacokinetics. Proc. Natl. Acad. Sci. 112 (19), 5997–6002. doi: 10.1073/pnas.1408766112 25918417 PMC4434771

[B56] Shields R. L. Lai J. Keck R. O'Connell L. Y. Hong K. Meng Y. G.. (2002). Lack of fucose on human IgG1 n-linked oligosaccharide improves binding to human FcγRIII and antibody-dependent cellular toxicity. J. Biol. Chem. 277 (30), 26733–26740. doi: 10.1074/jbc.M202069200 11986321

[B57] Simister N. E. (2003). Placental transport of immunoglobulin G. Vaccine Netherlands 21 (24), 3365–3369. doi: 10.1016/s0264-410x(03)00334-7 12850341

[B58] Stelter S. Paul M. J. Teh A. Y. Grandits M. Altmann F. Vanier J.. (2020). Engineering the interactions between a plant-produced HIV antibody and human fc receptors. Plant Biotechnol. J. 18 (2), 402–414. doi: 10.1111/pbi.13207 31301102 PMC6953194

[B59] Strasser R. Stadlmann J. Schähs M. Stiegler G. Quendler H. Mach L.. (2008). Generation of glyco-engineered nicotiana benthamiana for the production of monoclonal antibodies with a homogeneous human-like n-glycan structure. Plant Biotechnol. J. 6 (4), 392–402. doi: 10.1111/j.1467-7652.2008.00330.x 18346095

[B60] Tam S. H. McCarthy S. G. Brosnan K. Goldberg K. M. Scallon B. J. (2013). Correlations between pharmacokinetics of IgG antibodies in primates vs. FcRn-transgenic mice reveal a rodent model with predictive capabilities. mAbs 5 (3), 397–405. doi: 10.4161/mabs.23836 23549129 PMC4169033

[B61] Teh A. Y. Maresch D. Klein K. Ma J. K. (2014). Characterization of VRC01, a potent and broadly neutralizing anti-HIV mAb, produced in transiently and stably transformed tobacco. Plant Biotechnol. J. 12 (3), 300–311. doi: 10.1111/pbi.12137 24256218 PMC4112721

[B62] Tudor D. Yu H. Maupetit J. Drillet A. S. Bouceba T. Schwartz-Cornil I.. (2012). Isotype modulates epitope specificity, affinity, and antiviral activities of anti-HIV-1 human broadly neutralizing 2F5 antibody. Proc Natl Acad Sci U S A. 109 (31), 12680–12685. doi: 10.1073/pnas.1200024109 22723360 PMC3412007

[B63] UNAIDS (2019). 2018 global hiv statistics’. Unaids, 1–6.

[B64] UNAIDS (2021). Global HIV statistics. Fact Sheet, 1–3.

[B65] Valente D. Mauriac C. Schmidt T. Focken I. Beninga J. Mackness B.. (2020). Pharmacokinetics of novel fc-engineered monoclonal and multispecific antibodies in cynomolgus monkeys and humanized FcRn transgenic mouse models. MAbs 12 (1), 1829337. doi: 10.1080/19420862.2020.1829337 33079615 PMC7587234

[B66] Vidarsson G. Dekkers G. Rispens T. (2014). IgG subclasses and allotypes: From structure to effector functions. Front. Immunol. 5, 520. doi: 10.3389/fimmu.2014.00520 25368619 PMC4202688

[B67] Ward B. J. Séguin A. Couillard J. Trépanier S. Landry N. (2021). Phase III: Randomized observer-blind trial to evaluate lot-to-lot consistency of a new plant-derived quadrivalent virus like particle influenza vaccine in adults 18-49 years of age. Vaccine Netherlands 39 (10), 1528–1533. doi: 10.1016/j.vaccine.2021.01.004 33581920

[B68] Wei X. Decker J. M. Wang S. Hui H. Kappes J. C. Wu X.. (2003). Antibody neutralization and escape by HIV-1. Nature Engl. 422 (6929), 307–312. doi: 10.1038/nature01470 12646921

[B69] World Health Organization (2019)HIV drug resistance report. Available at: http://scholar.google.com/scholar?hl=en&btnG=Search&q=intitle:Who+hiv+drug+resistance+report+2012#5.

[B70] Wu X. Yang Z. Y. Li Y. Hogerkorp C. M. Schief W. R. Seaman M. S.. (2010). Rational design of envelope identifies broadly neutralizing human monoclonal antibodies to HIV-1. Science 329 (5993), 856–861. doi: 10.1126/science.1187659.Rational 20616233 PMC2965066

[B71] Yogo R. Yamaguchi Y. Watanabe H. Yagi H. Satoh T. Nakanishi M.. (2019). The Fab portion of immunoglobulin G contributes to its binding to Fcγ receptor III. Sci Rep. 9 (1), 11957. doi: 10.1038/s41598-019-48323-w 31420591 PMC6697678

[B72] Zalevsky J. Chamberlain A. K. Horton H. M. Karki S. Leung I. W. Sproule T. J.. (2010). Enhanced antibody half-life improves *in vivo* activity. Nat Biotechnol. 28 (2), 157–9. doi: 10.1038/nbt.1601 20081867 PMC2855492

[B73] Zeitlin L. Pettitt J. Scully C. Bohorova N. Kim D. Pauly M.. (2011). Enhanced potency of a fucose-free monoclonal antibody being developed as an Ebola virus immunoprotectant. Proc Natl Acad Sci U S A. 108 (51), 20690–20694. doi: 10.1073/pnas.1108360108 22143789 PMC3251097

