# Supplementary material for: Improving the efficacy of plant-made anti-HIV monoclonal antibodies for clinical use
Source: Front Plant Sci. 2023 Feb 27;14:1126470. doi: 10.3389/fpls.2023.1126470 (PMC10009187; doi:10.3389/fpls.2023.1126470)
Supplement: Supplementary Table 1 — Primers used in site-directed mutagenesis to introduce M252Y/S254T/T256E (YTE) or M428L/N434S (LS) mutation in heavy (γ) chain Fc regions of VRC01 or 3BNC117/10-1074. Nucleotides in bold indicate changes in the sequence. A different primer pair was chosen for VRC01 as a plasmid was already available and the nucleotide sequence of the Fc region -while maintaining the same amino acid composition- differed slightly to the one used for 3BNC117 and 10-1074. [file DataSheet_1.docx]

**Table S1.** Primers for site directed mutagenesis

| **Construct** | **Forward primer** | **Reverse Primer** |
| --- | --- | --- |
| VRC01 YTE | 5’CCTAAGGATACCCTT**TAT**ATT**ACT**CGG**GAA**CCTGAGGTTACCTGC-3’ | 5’GCAGGTAACCTCAGG**TTC**C**CGA**GTA**ATA**TAAAGGGTATCCTTAGG-3’ |
| VRC01 LS | 1: 5’CTCTTGCTCTGTT**CTG**CATGAGGCTCTG-3’  2: 5’GAGGCTCTGCAT**AGT**CACTACACCCAG-3’ | 1: 5’CAGAGCCTCATG**CAG**AACAGAGCAAGAG-3’  2: 5’CTGGGTGTAGTG**ACT**ATGCAGAGCCTC-3’ |
| 3BNC117 & 10-1074 YTE | 5’CCAAAGGATACTCTC**TAT**ATC**ACT**CGG**GAA**CCAGAGGTTACATGC-3’ | 5’GCATGTAACCTCTGG**TTC**CCG**AGT**GAT**AT**AGAGAGTATCCTTTGG-3’ |
| 3BNC117 & 10-1074 LS | 1: 5’CTTGCTCTGTT**CTG**CATGAGGCTCTCC-3’  2: 5’GAGGCTCTCCAC**TCC**CACTACACTCAGAAG-3’ | 1: 5’GGAGAGCCTCATG**CAG**AACAGAGCAAG-3’  2: 5’CTTCTGAGTGTAGTG**GGA**GTGGAGAGCCTC-3’ |

**Table S2.** Binding affinity to HIV-1 UG37 gp140

| **bNAb** | **Affinity K_D_ [M]** | **k_a_ [1/Ms]** | **k_d_ [1/s]** |
| --- | --- | --- | --- |
| VRC01 HEK | 7.23*10^-10^ | 1.77*10^4^ | 1.27*10^-5^ |
| VRC01 HC | 29.2*10^-10^ | 0.57*10^4^ | 1.66*10^-5^ |
| VRC01 YTE | 42.1*10^-10^ | 0.62*10^4^ | 2.60*10^-5^ |
| VRC01 LS | 43.9*10^-10^ | 0.57*10^4^ | 2.51*10^-5^ |
| 3BNC117 HEK | 6.69*10^-9^ | 2.9*10^3^ | 1.94*10^-5^ |
| 3BNC117 HC | 4.02*10^-9^ | 4.35*10^3^ | 1.75*10^-5^ |
| 3BNC117 YTE | 3.04*10^-9^ | 9.32*10^3^ | 2.83*10^-5^ |
| 3BNC117 LS | 3.96*10^-9^ | 9.899*10^3^ | 3.92*10^-5^ |

**Table S3.** HIV-1 IC50 VRC01 (top) 3BNC117 (bottom)

**VRC01**

|  |  |  | **IC_50_ (ng/mL)** | |  |
| --- | --- | --- | --- | --- | --- |
| **HIV-1 Pseudovirus** | **Clade** | **HEK** | **HC ∆XF** | **YTE ∆XF** | **LS ∆XF** |
| BaL.26 | C | 1870 ± 140 | 1710 ± 680 | 1880 ± 900 | 1860 ± 103 |
| CAAN5342 | B |  | 0.24 | 0.072 | 0.051 |
| SC422-661.8 | B |  | 0.029 | 0.005 | 0.015 |
| ZM53M.PB12 | C |  | 0.103 | 0.058 | 0.082 |
| ZM109F.PB4 | C |  | 0.011 | 0.003 | 0.010 |

**3BNC117**

|  |  |  | **IC_50_ (ng/mL)** | |  |
| --- | --- | --- | --- | --- | --- |
| **HIV-1 Pseudovirus** | **Clade** | **HEK** | **HC ∆XF** | **YTE ∆XF** | **LS ∆XF** |
| BaL.26 | C | 0.14 ± 0.05 | 0.10 ± 0.05 | 0.21 ± 0.07 | 0.17 ± 0.04 |
| CAAN5342 | B | 48.24 | 87.29 | 52.16 | 38.19 |
| SC422-661.8 | B | 3.21 | 4.98 | 5.74 | 2.79 |
| ZM53M.PB12 | C | 26.05 | 35.00 | 41.04 | 28.51 |
| ZM109F.PB4 | C | 25.49 | 38.83 | 18.12 | 24.72 |

**Table S4.** Binding affinity of 10-1074 variants to HIV-1 UG37 gp140

| **bNAb** | **Affinity K_D_ [M]** | **k_a_ [1/Ms]** | **k_d_ [1/s]** |
| --- | --- | --- | --- |
| 10-1074 CHO | 8.24*10^-10^ | 1.43*10^4^ | 1.18*10^-5^ |
| 10-1074 HC ΔXF | 18.7*10^-10^ ± 7.71*10^-10^ | 1.18*10^4^ ± 0.352*10^4^ | 2.04*10^-5^ ± 0.395*10^-5^ |
| 10-1074 YTE ΔXF | 22.2*10^-10^ ± 28.6*10^-10^ | 1.39*10^4^ ± 0.535*10^4^ | 2.19*10^-5^ ± 2.44*10^-5^ |

**Table S5.** Area under the curve (AUC) (%*h) for all *in vivo* half-life experiments with variants of 10-1074

| **Experiment no.** | **10-1074 CHO** | **10-1074 HC ΔXF** | **10-1074 YTE ΔXF** |
| --- | --- | --- | --- |
| 1 |  | 64.5 ± 13.8 (n=3) | 120.4 ± 8.7 (n=3) |
| 2 |  | 82.9 ± 26.9 (n=4) | 81.2 ± 6.9 (n=4) |
| 3 |  | 104.4 ± 27.3 (n=4) | 134.4 ± 49 (n=5) |
| 4 | 115.5 ± 32.6 (n=3) | 59.2 ± 2.1 (n=2) |  |
| Mean ± SD  Individual animals | 115.5 ± 32.6  n=3 | 81.6 ± 23.0  n=13 | 113.2 ± 38.6  n=12 |
|  |  |  |  |

|  | **10-1074** | | | **VRC01** | | | | **3BNC117** | | | |
| --- | --- | --- | --- | --- | --- | --- | --- | --- | --- | --- | --- |
|  | **HC ΔXF** | **YTE ΔXF** | **CHO** | **HC ΔXF** | **YTE ΔXF** | **LS DXF** | **HEK** | **HC ΔXF** | **YTE ΔXF** | **LS ΔXF** | **HEK** |
| **Yield (post-purification*) (mg/kg_Fresh weight_)** | 47.0 ± 10.8 | 12.6 ± 0.2 | nd | 65 ± 8 | 39 ± 33 | 33 ± 16 | nd | 51 ± 21 | 31 ± 17 | 38 ± 27 | nd |
| **gp140 affinity KD [M]** | 18.7*10^-10^ | 22.2*10^-10^ | 8.24*10^-10^ | 29.2*10^-10^ | 42.1*10^-10^ | 43.9*10^-10^ | 7.23*10^-10^ | 4.02*10^-9^ | 3.04*10^-9^ | 3.96*10^-9^ | 6.69*10^-9^ |
| **BaL.26 (IC50) [ng/ml]** | 93 ± 15 | 74 ± 15 | 81 ± 10 | 1710 ± 680 | 1880 ± 900 | 1860 ± 103 | 1870 ± 140 | 0.10 ± 0.05 | 0.21 ± 0.07 | 0.17 ± 0.04 | 0.14 ± 0.05 |
| **FcγRIIIa V158 affinity [M]** | 8.32*10^-8^ | 12.4*10^-8^ | 78*10^-8^ | 4.50*10^-8^ | 9.12*10^-8^ | 7.27*10^-8^ | 25.0*10^-8^ | 3.98*10^-8^ | 19.6*10^-8^ | 13.2*10^-8^ | 59.0*10^-8^ |
| **Transcytosis (Fold change at 10 ug input)** | 1 | 3.4 | 0.9 | 1.0 | 2.9 | 2.4 | 1.1 | 1.0 | 4.5 | 2.4 | 1.0 |
| **Pharmacokinetics (mean AUC)** | 81.6 ± 23.0 (n=13) | 113.2 ± 38.6 (n=12) | 115.5** ± 32.6 (n=3) | 47.0 ± 9.5 (n=5) | 72.5 ± 10.2 (n=4) | 69.1 ± 13.1 (n=4) | 135 ± 116.3 (n=6) | nd | nd | nd | nd |

**Table S6.** Summary table of results for all variants of the 3 bNAbs 10-1074, VRC01 and 3BNC117

*highest average yield achieved (10-1074 HC and YTE with addition of 0.01% polysorbate 80)

** from single experiment with one mouse showing a rise in serum levels after drop at two timepoints – this resulted in skewing of average AUC

**Figure S1**

**A**

|  | **Post-purification Yield [mg/kg fresh weight]** | | |
| --- | --- | --- | --- |
| **bNAb** | **HC ΔXF** | **YTE ΔXF** | **LS ΔXF** |
| VRC01 | 65 ± 8 | 39 ± 33 | 33 ± 16 |
| 3BNC117 | 51 ± 21 | 31 ± 17 | 38 ± 27 |

**B**

1 2 3 4

1: VRC01 ∆XF HC+LC

2: VRC01 ∆XF YTE+LC

3: VRC01 ∆XF LS+LC

4: VRC01 HEK

**Figure S1 (continued)**

**C**

**
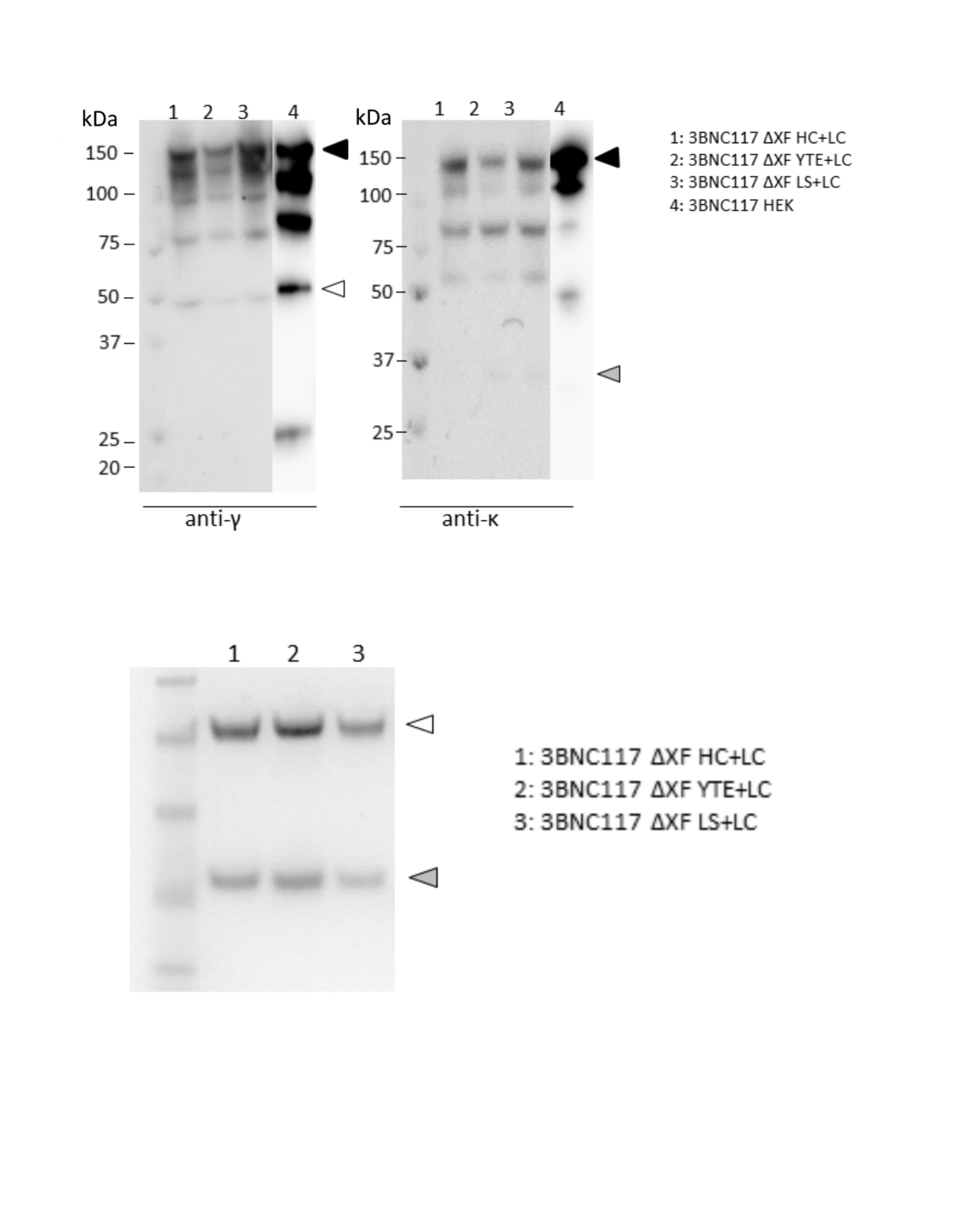
**

**D**

**
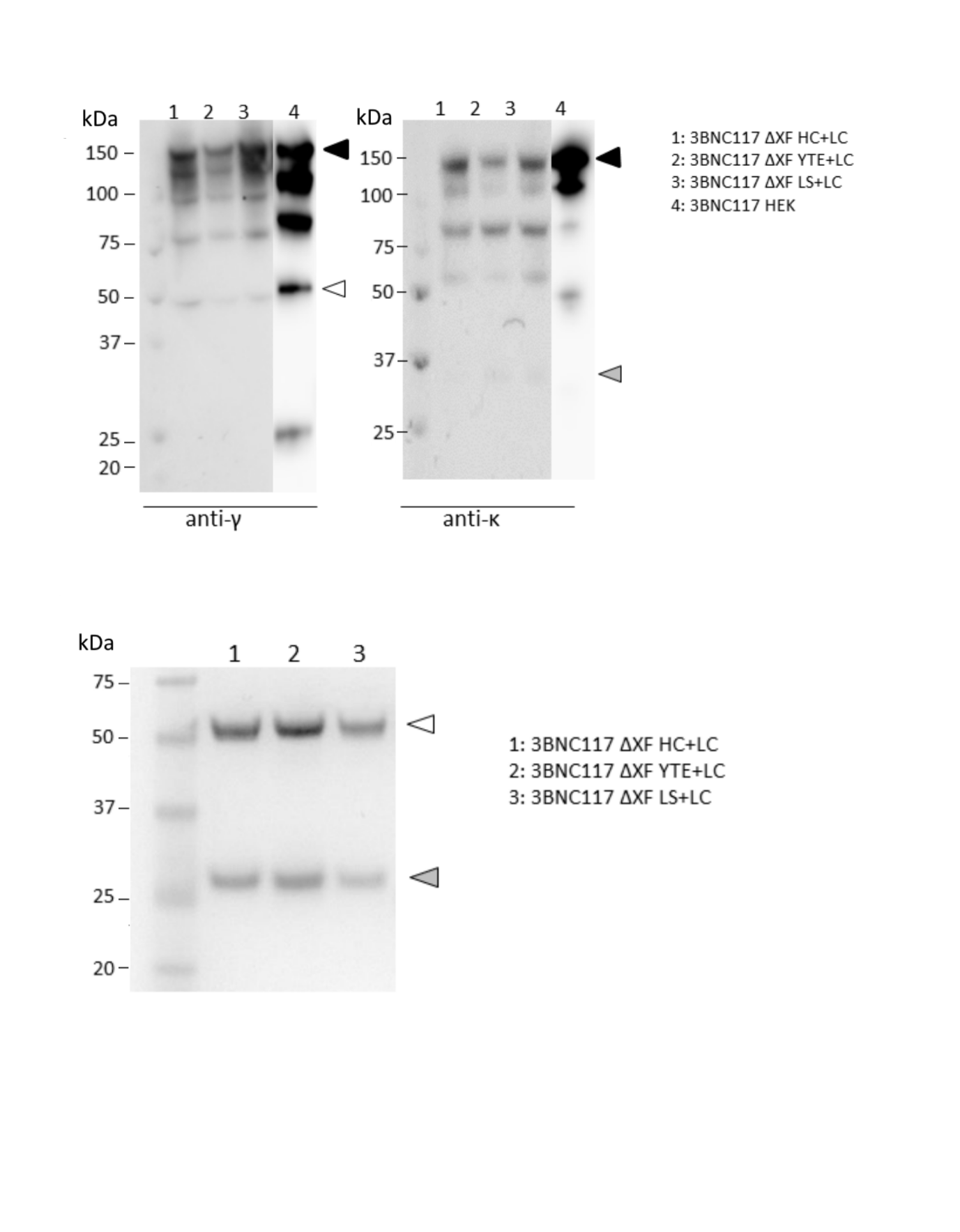
**

**Figure S2**

**A**

**
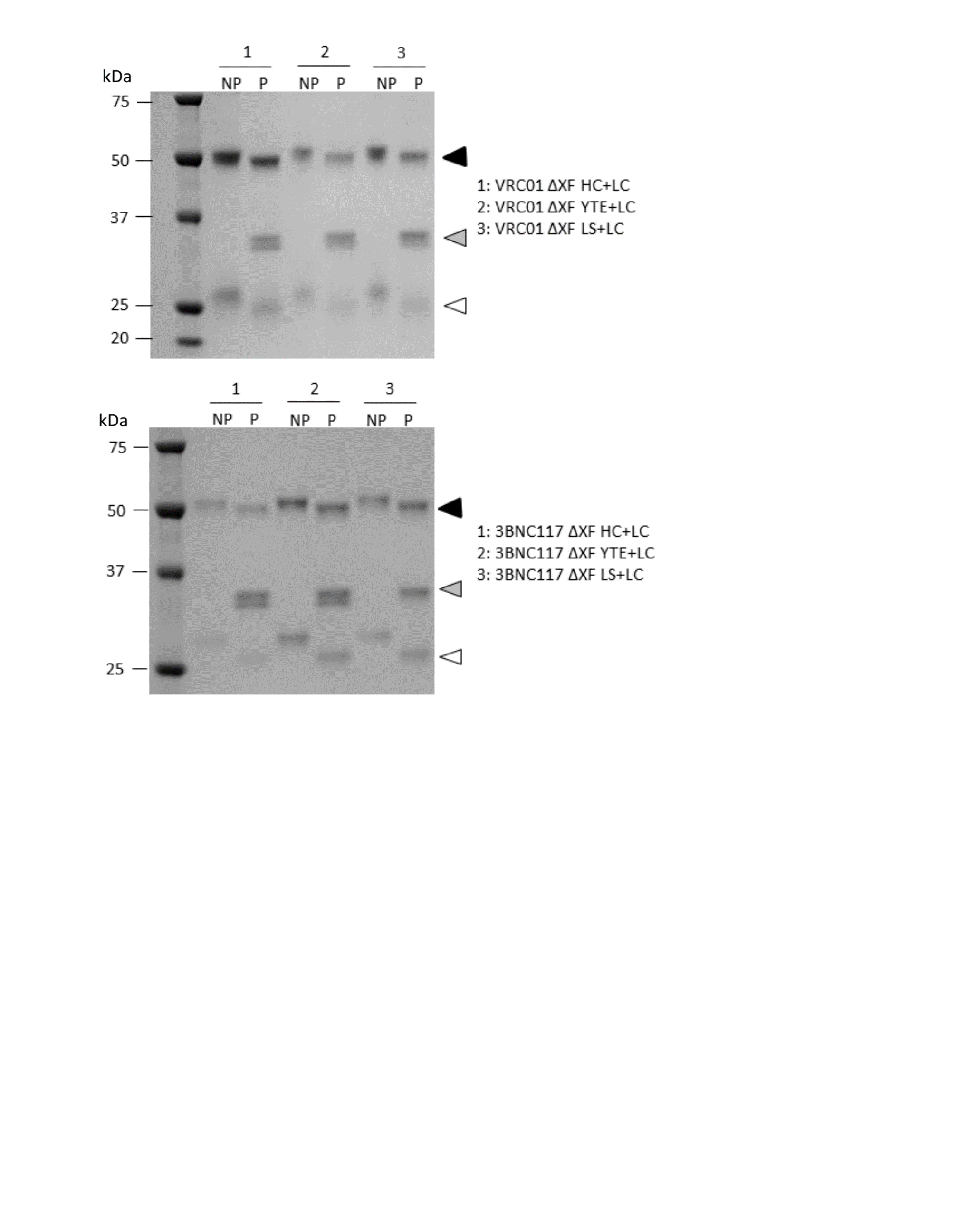
**

**B**

**
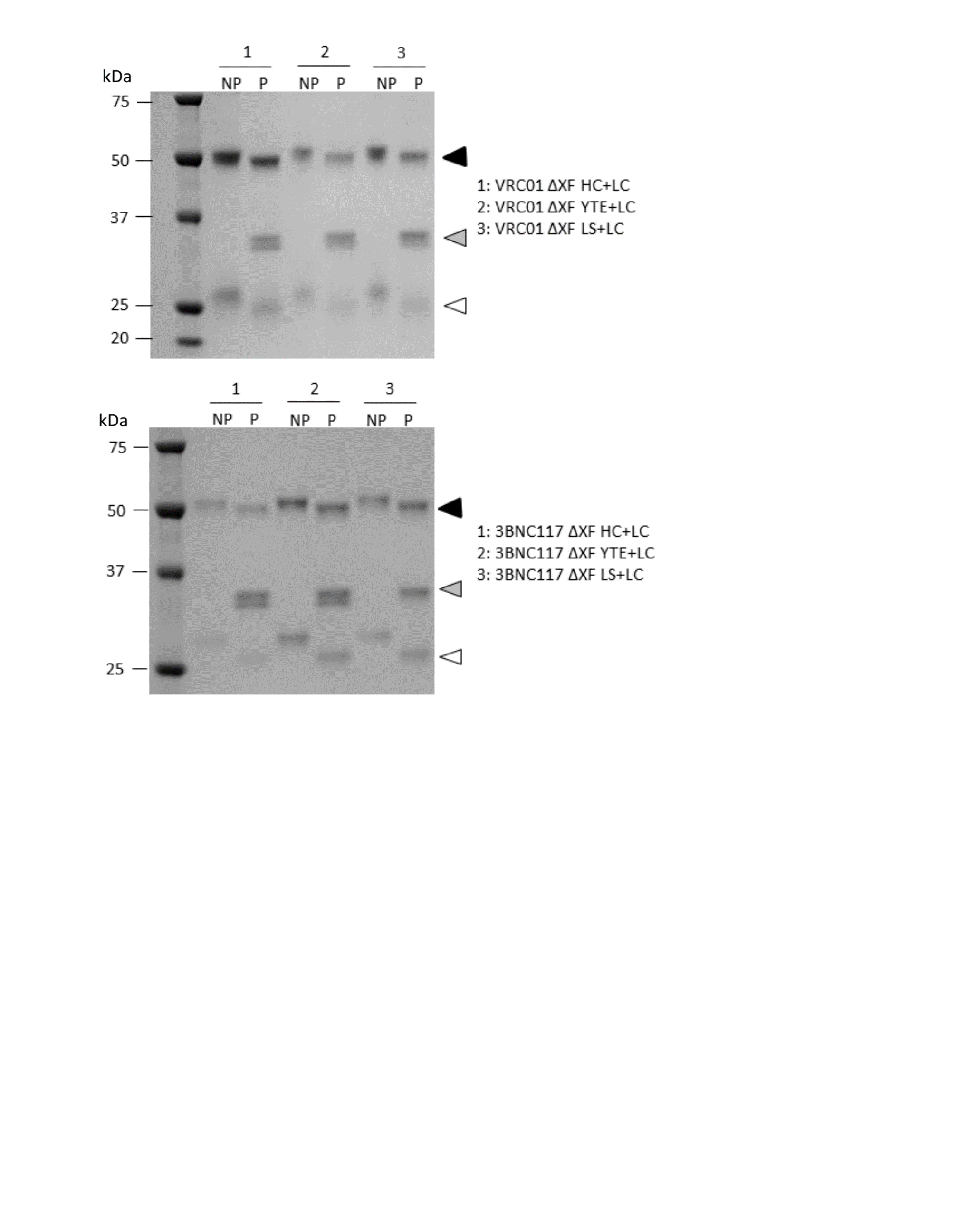
**

**Figure S3.**

**A**

| Sample | K_D_ [M] |
| --- | --- |
| VRC01 HEK | 25.0*10^-8^ |
| VRC01 ∆XF HC | 4.50*10^-8^ |
| VRC01 ∆XF YTE | 9.12*10^-8^ |
| VRC01 ∆XF LS | 7.27*10^-8^ |
| 3BNC117 HEK | 59.0*10^-8^ |
| 3BNC117 ∆XF HC | 3.98*10^-8^ |
| 3BNC117 ∆XF YTE | 19.6*10^-8^ |
| 3BNC117∆XF LS | 13.2*10^-8^ |

Binding affinity to FcγRIIIa V158

**Figure S3 (continued)**

**B**

**VRC01**

**
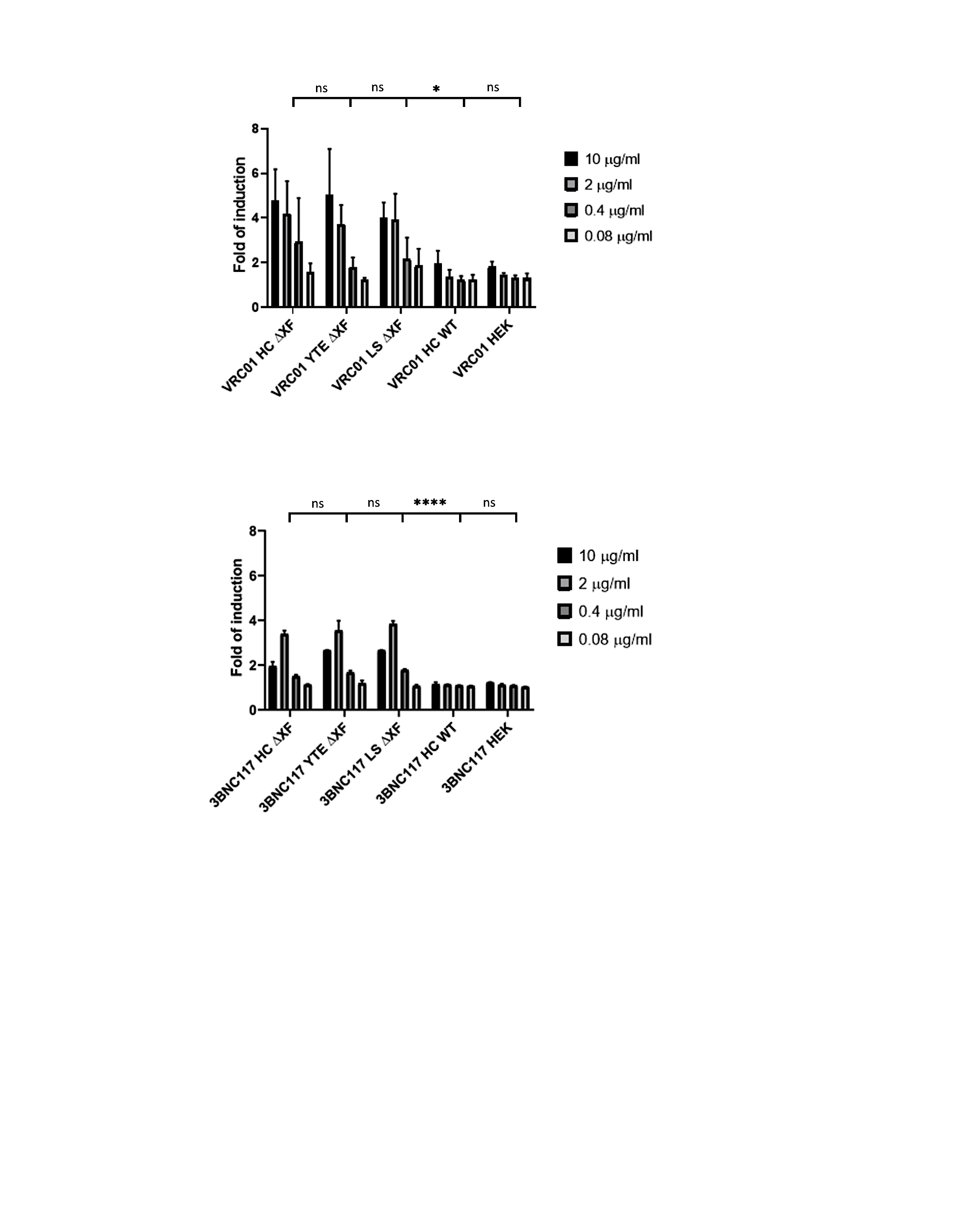
**

**3BNC117**

**Figure S4**

**A**

| bNAb | Affinity K_D_ [M] |
| --- | --- |
| VRC01 HEK | 18.79*10^-8^ |
| VRC01 ∆XF HC | 45.4*10^-8^ |
| VRC01 ∆XF YTE | 7.01*10^-8^ |
| VRC01 ∆XF LS | 4.54*10^-8^ |
| 3BNC117 HEK | 14.2*10^-8^ |
| 3BNC117 ∆XF HC | 20.8*10^-8^ |
| 3BNC117 ∆XF YTE | 8.4*10^-8^ |
| 3BNC117 ∆XF LS | 9.47*10^-8^ |

**B**

**VRC01**


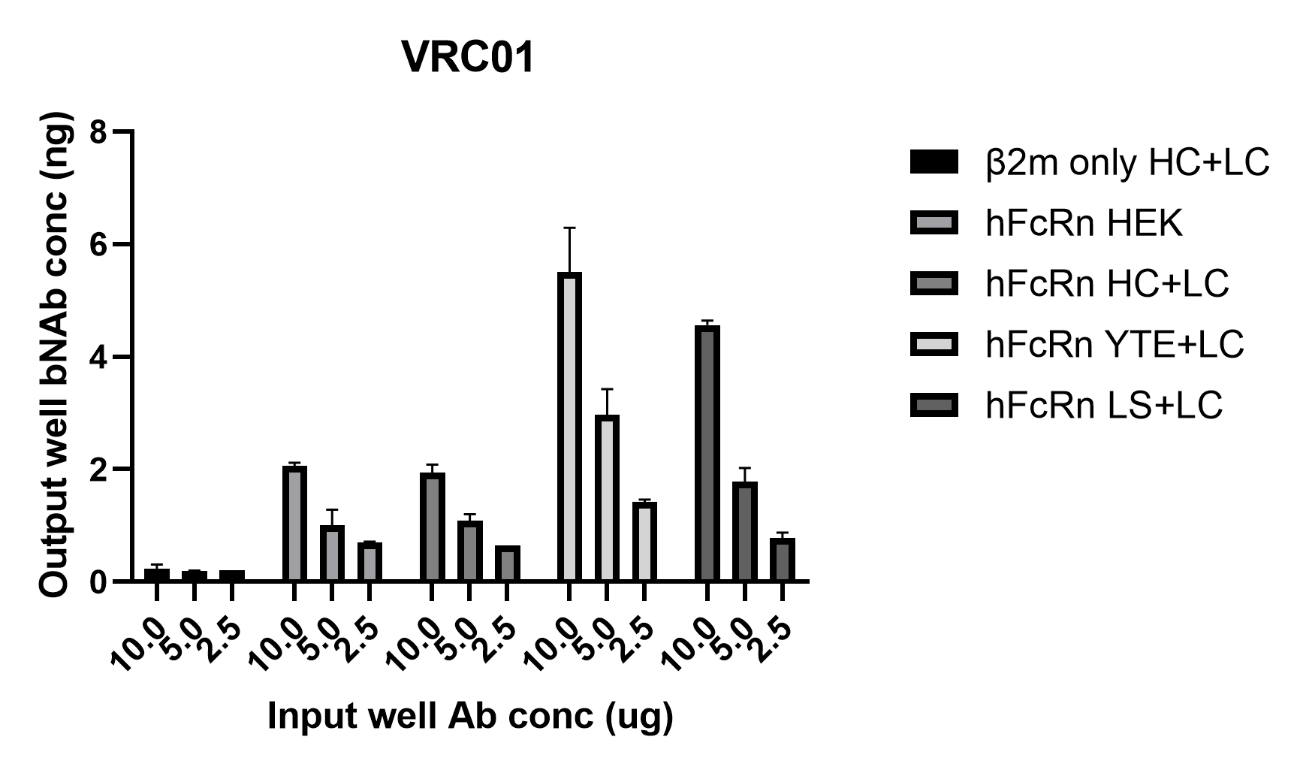


**Figure S4 (continued)**

**C**

**3BNC117**

**
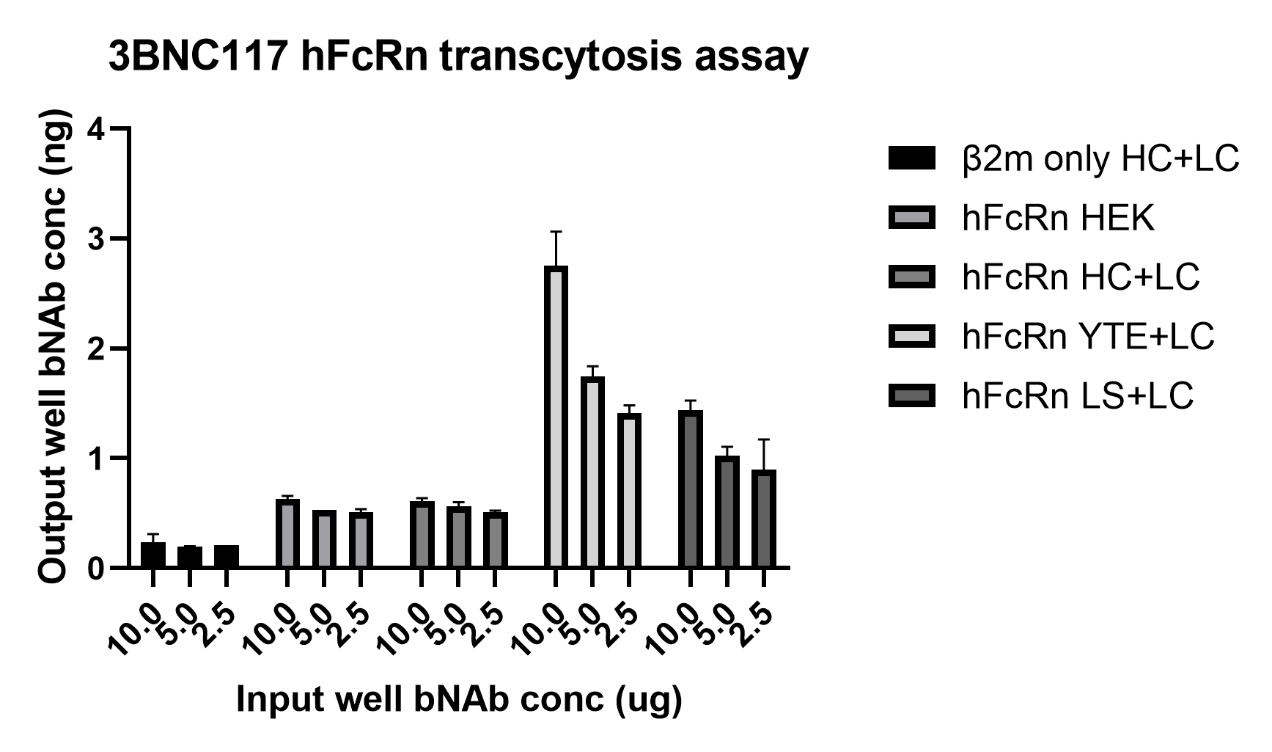
**

**Figure S5**

**A**


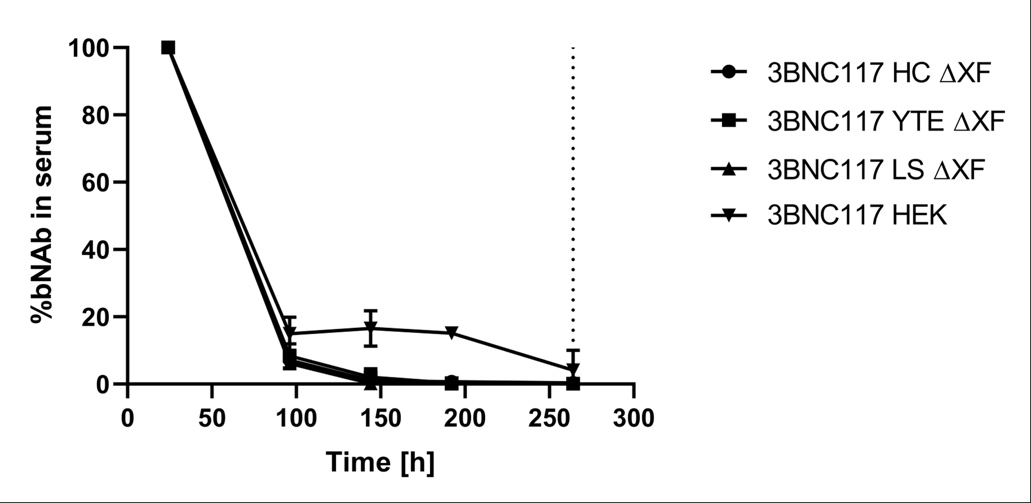
 **
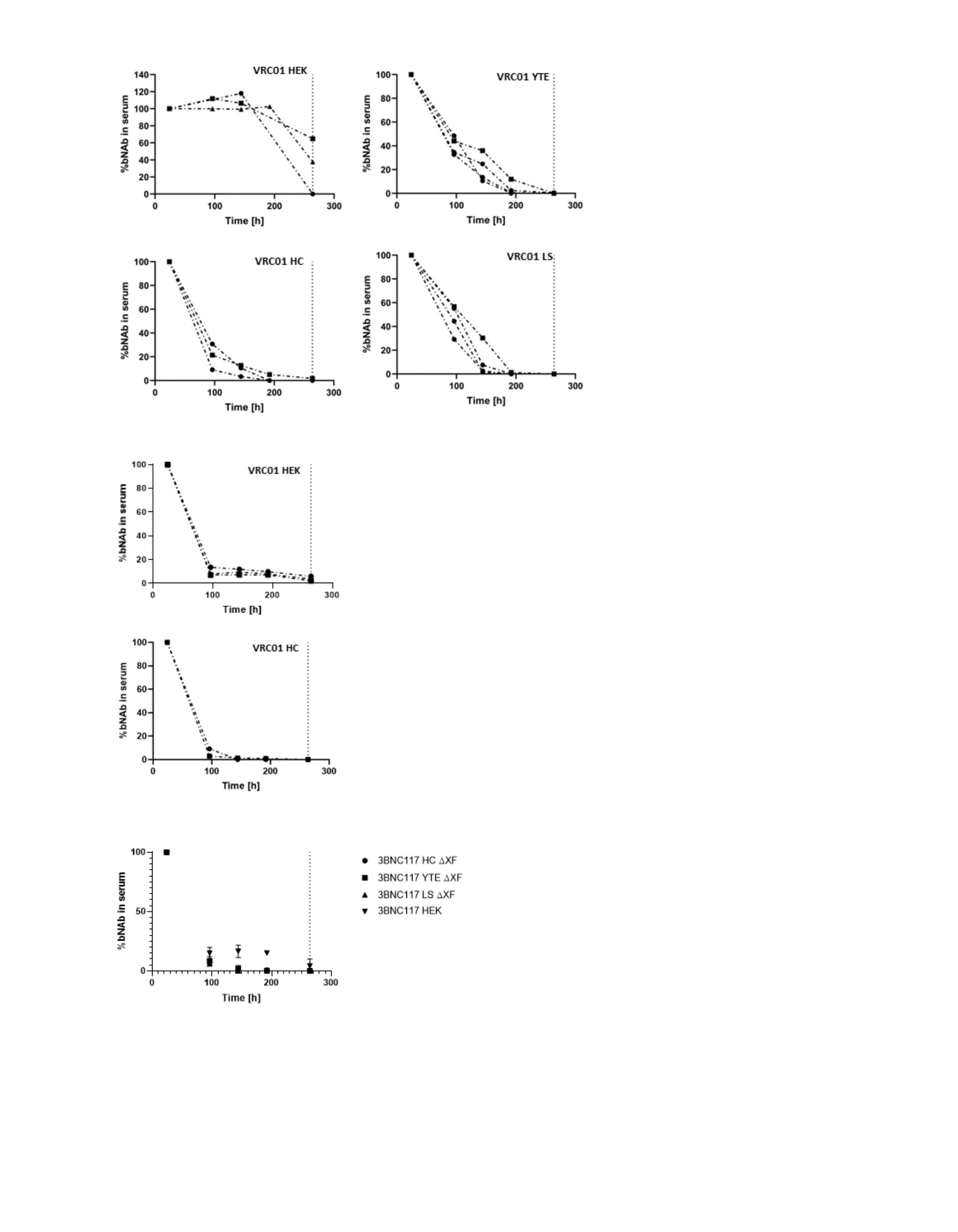
**

**B**


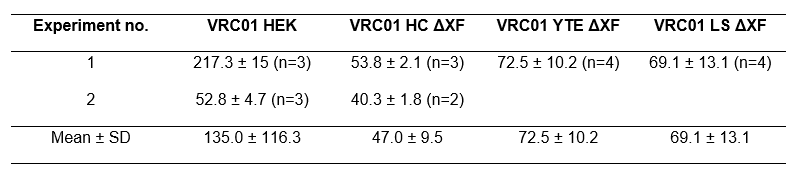


**B**

**Figure S6. Relative anti-hIgG murine IgM levels in serum at final timepoint**

**Figure S7. Linear and mixed-mode pharmacokinetics of 10-1074 variants**

**
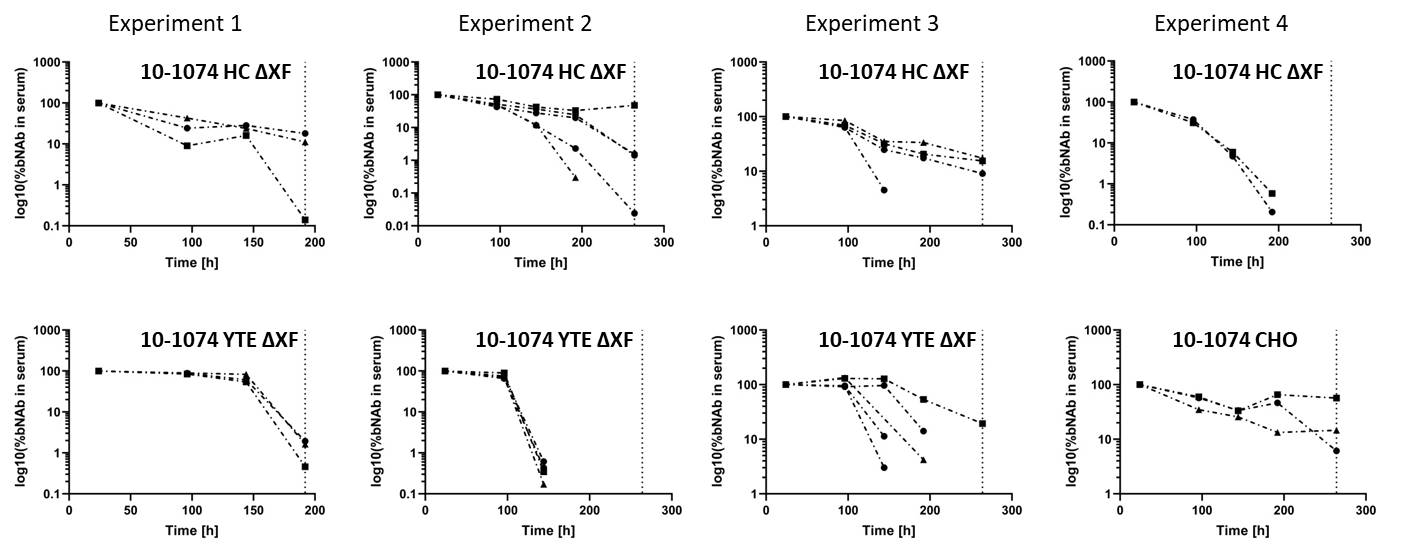
**

Supplementary Methods

**Methods S1. IgM ELISA**

A murine IgM anti-human IgG ELISA was used to determine immunogenicity of the bNAbs in vivo. 96 flat-bottom well immunosorbent plates were coated with 5 μg/mL human IgG1 λ (Sigma Aldrich, USA) in PBS 7.4 and incubated overnight at 4 °C. All blocking and washing steps were performed as described for all other ELISAs performed. Serum was applied in a 1 in 100 dilution in duplicates and further diluted 1:1 using blocking buffer. Plates were incubated overnight at 4 °C. After washing, an anti-murine IgM antibody (1 in 1000) in blocking buffer was applied and plates were incubated for 1 h at 37 °C. For detection 100 μL of SIGMAFAST™ OPD (Sigma Aldrich, USA) were added to each well. A492 was measured using a Tecan Sunrise plate reader.
